# Machine learning in diagnostic support in medical emergency departments

**DOI:** 10.1038/s41598-024-66837-w

**Published:** 2024-08-02

**Authors:** Claus Lohman Brasen, Eline Sandvig Andersen, Jeppe Buur Madsen, Jens Hastrup, Henry Christensen, Dorte Patuel Andersen, Pia Margrethe Lind, Nina Mogensen, Poul Henning Madsen, Anne Friesgaard Christensen, Jonna Skov Madsen, Ejler Ejlersen, Ivan Brandslund

**Affiliations:** 1 Department of Biochemistry and Immunology, Lillebaelt Hospital, University Hospital of Southern Denmark, Beriderbakken 4, 7100 Vejle, Denmark; 2 Faculty of Health Sciences, Department of Regional Health Research, University of Southern Denmark, Campusvej 55, 5230 Odense M, Denmark; 3 Department of Emergency, Kolding Hospital, Lillebaelt Hospital, University Hospital of Southern Denmark, Sygehusvej 24, 6000 Kolding, Denmark; 4 Department of Medicine, Kolding Hospital, Lillebaelt Hospital, University Hospital of Southern Denmark, Sygehusvej 24, 6000 Kolding, Denmark; 5 Emergency, Acute Care and Trauma Centre, Odense University Hospital, J. B. Winsløws Vej 4, 5000 Odense, Denmark; 6 Department of Medicine, Vejle Hospital, Lillebaelt Hospital, University Hospital of Southern Denmark, Beriderbakken 4, 7100 Vejle, Denmark

**Keywords:** Diagnosis, Diseases

## Abstract

Diagnosing patients in the medical emergency department is complex and this is expected to increase in many countries due to an ageing population. In this study we investigate the feasibility of training machine learning algorithms to assist physicians handling the complex situation in the medical emergency departments. This is expected to reduce diagnostic errors and improve patient logistics and outcome. We included a total of 9,190 consecutive patient admissions diagnosed and treated in two hospitals in this cohort study. Patients had a biochemical workup including blood and urine analyses on clinical decision totaling 260 analyses. After adding nurse-registered data we trained 19 machine learning algorithms on a random 80% sample of the patients and validated the results on the remaining 20%. We trained algorithms for 19 different patient outcomes including the main outcomes death in 7 (Area under the Curve (AUC) 91.4%) and 30 days (AUC 91.3%) and safe-discharge(AUC 87.3%). The various algorithms obtained areas under the Receiver Operating Characteristics -curves in the range of 71.8–96.3% in the holdout cohort (68.3–98.2% in the training cohort). Performing this list of biochemical analyses at admission also reduced the number of subsequent venipunctures within 24 h from patient admittance by 22%. We have shown that it is possible to develop a list of machine-learning algorithms with high AUC for use in medical emergency departments. Moreover, the study showed that it is possible to reduce the number of venipunctures in this cohort.

## Introduction

Diagnosing patients in medical emergency settings can be challenging due to the fast-paced environment, limited staff resources, and a large number of patients presenting with diverse disease presentations which increase the risk of diagnostic errors^1–3^. The strain on emergency departments is predicted to increase as many countries experience an ageing population. For instance, in Denmark, projections show a 25% increase in individuals aged 66 years and above and a reduction of 9.4% in the adult population below 66 years of age by year 2040^4^. Kachman et al.^5^ has described in detail which areas in emergency care could be alleviated by machine learning. One area would be interpreting diagnostic biomarker results, which are getting cheaper and more easily available making the diagnostic process increasingly complex. Indeed there are examples of machine learning algorithms outperforming humans in interpreting paraclinical data from for example pathology^6^ and radiology^7^. This complexity places further strain on the clinical staff which in turn may increase error-rates. Introducing machine learning algorithms as expert-support systems to alleviate the workload and reduce error-rates could be a viable way forward. Feretzakis et al. developed a machine learning algorithm to predict hospital admission on patients arriving in the emergency department using standard available biomarker data^8^. Others have studied other outcomes such as readmissions and found that it is possible to train algorithms for this outcome with an area under the curve (AUC) of 0.82^9^. Predicting death in a certain time period following admission has been studied by several groups of which Look et al. has improved the SERP algorihms and reached an AUC of 0.893 for 7 days and 0.890 for 30 days^10^ still not a perfect algorithm and leaving room for more useful features. Instead of studying one outcome, Buergel et al.^11^ has shown that this is possible on a large cohort to identify multiple diseases using metabolomics, although not on emergency patients and not with quick delivery of results. The objective of this study was to train and validate machine learning algorithms for predicting a list of common medical conditions in a medical emergency setting using an expanded but possible list of clinical and laboratory data. The secondary aim was to study the effect of using an expanded list of biomarkers on the number of subsequent venipunctures within the following 24 h.

In order to effectively train, validate and apply machine learning algorithms that offers a value-addition in clinical effect, it is essential to identify the variables and clinical data that adds value. Since research on using diagnostic tests on large cohorts is often limited in scope and incremental in terms of analyses tested, this approach is insufficient. Therefore, we adopted a different approach by conducting a comprehensive range of biochemical analyses upon admission. This allowed us to train machine learning algorithms to diagnose and predict prognosis for patients often before the physician even see the patient, and identify the variables of value for medical emergency patients. To ensure clinical feasibility, we focused on analyses that a hospital’s clinical chemistry laboratory could deliver within one hour.

## Materials and methods

### Patients

All patients arriving at the medical emergency departments of Kolding and Vejle hospitals in the period Sep-2020 to Apr-2021 were triaged by a trained nurse and vital parameters were recorded. All admitted patients were included consecutively aside from a 13-days Christmas break in this retrospective cohort study. Kolding Hospital is the major emergency hospital receiving more severe cases than Vejle Hospital. The medical emergency departments handle all adult medical emergencies except if the main emergency medical issue is related to heart disease, neurological disease, oncology or obstetrics and gynecology. In case of possible acute surgery (in case of acute abdomen) or suspected covid19, patients are directed to Kolding Hospital. All admitted patients had a venipuncture performed by a trained biotechnologist and a pre-specified set of samples drawn immediately and analyses performed including an electrocardiogram (ECG) (see Suppl. Table S1). Urine samples were taken on clinical decision, in which case a pre-specified set of analyses were performed (see Suppl. Table S1). Generally, all analyses were performed within 60–80 min, however, four analyses were performed in one hospital only and hence took longer, but all analyzes could be set up to run in approximately 60 min. All physicians received the results from the standard list of required blood tests (see Table S4) and requested urine analyses together with the ECG. In case a physician ordered additional analyses, these were supplied from the already analyzed results. Panic limits are used in the hospital for some biomarkers for which extreme values requires urgent attention. Results that exceed decided critical limits called “panic limits” are handled in a way that secures fast response. Results were automatically sent to the physician in case a panic limit was exceeded for the analyses listed in Table S3. Data from patients with urine analysis and urine cultivation results have been compared in a previous article^12^.

#### Venipuncture

The blood sampling was performed by venipuncture using a butterfly needle (Safety blood Collection set, Greiner Bio One AG, Kremsmünster, Austria). Nine tubes were sampled from each patient at admittance resulting in a total volume of 38 mL blood. Approximately 22% of emergency medical patients are normally sampled for a minimum of 38 mL in the first 24 h in the hospital. Special tubes were used for lactate, glucose and blood gas. Air bubbles were immediately vented from the blood gas tube (Sarstedt AG & Co. KG, Nümbrecht, Germany). Gas samples were hand carried and analysed by ABL(Acid Base Laboratory, Radiometer A/S Copenhagen, Denmark) while the other samples were transported to the laboratory using Tempus600 pressurized tube system (Sarstedt ApS, Bording, Denmark).

#### Biochemical analyses

All 260 analyses were performed on instruments (see Table 1) in the two DS/EN ISO 15189 accredited biochemical hospital laboratories or on decentralized ABL instruments. All equipment is CE-marked and the majority of analyses are accredited (Danish Accreditation Fund, DANAK). Vasopressin-neurophysin 2-copeptin(126–164), Corticotropine, Parathyrin hormone and Proadrenomedullin (45–92) were performed in one hospital lab only and done on plasma frozen at minus 80 degrees Celsius for up to 4 days until analysis, following the exact same procedure concerning freezing and thawing for both hospitals. All other analyses were performed in both hospital laboratories using the normal stat-setup. The list of biomarkers (see Table S1) used in this study were selected based on the current state of technology’s ability to deliver results for potentially relevant biomarkers within 60 min from venipuncture as relevant for medical emergency patient evaluation.

**Table 1:** Data sources, feature packages and instruments used.

Feature package	Instrument	Number of features (variables)	Average availability, %
ECG	MAC 5500, GE Healthcare	26	95.2%
Blood	A range of instruments:	64	99.0%
	Cobas 8000, Roche		
	CS5100, Siemens		
	Kryptor, Thermo Fisher		
	Tosoh G8, Tosoh Bioscience		
Blood(expensive)	Cobas 8000, Roche / Kryptor, Thermo Fisher	7	99.0%
Blood(hematology)	XN 9000/DI60, Sysmex	18	99.0%
Blood(hematology, research)	XN 9000/DI60, Sysmex	52	97.7%
Blood(hematology, correlated)	XN 9000/DI60, Sysmex	20	97.7%
Blood(gas tension and acid/base)	ABL 800, Radiometer	18	96.9%
Blood(gas tension and acid/base, research)	ABL 800, Radiometer	5	96.0%
Blood(gas tension and acid/base, correlated)	ABL 800, Radiometer	10	96.1%
Urine(biochem + Flow)	UF5000, Sysmex	2	38.2%
	Cobas 8000, Roche	2	38.5%
Urine(Flow, research)	UF5000, Sysmex	28	37.3%
Urine(Flow, correlated)	UF5000, Sysmex	1	37.3%
Urine(dipsticks)	Urine sticks, Clinitek, Siemens	7	53.0%
Nurse(physical measures)	Measures by nurse	9	87.2%
Nurse(patient scores)	Measures by nurse	4	92.5%

The rightmost column shows the average data availability for each package.

#### Registry data on diagnoses and death

Diagnosis data were used both as input (historical) and outcome (discharge diagnoses) data. We retrieved data on disease on all patients from both the regional clinical database and a national database (Esundhed, Danish Health Data Authority) in order to obtain both relevant historical disease status and precise admission data on all patients; to calculate 5 year Charlson Index^13,14^ and to obtain the diseases recorded by the physicians after discharge on the actual admission and 30 days post-admission. Charlson Index was used as a marker for preexisting medical disease in each patient. The regional database is more precise and is used for specific date and time and for registering patients that change between departments. The national database is used to supply with data from patient record from other regions. International hospital admissions are not covered. Table S2 describes the diagnosis classifications in the study. Prior occurrence of the same diagnosis group as the target outcome was included as an input variable together with the number of days since last occurrence of the diagnosis group for each patient for training of the algorithms (e.g. the risk of pneumonia could be increased in case the patient has had pneumonia prior to the current admission). For future use of diagnosis data it is possible to extract data every night from the regional database making it readily available to the algorithms.

Both primary- and secondary-diagnoses were retrieved and used as appropriate for acute and chronic diseases (all diagnoses within 30 days of admittance). Date of death was received from the national Civil Registration System. We investigated the top15-diagnosis groups and 5 outcomes not based on specific diagnoses (die within 7 days on admittance, die within 30 days of admittance, respirator treatment, intensive care treatment and safe-discharge). For the purpose of our study, we defined safe-discharge as patients admitted for a maximum of 24 h, not dying within 30 days of admission and not being readmitted (all Danish hospitals included) within 30 days. Although electrolyte-problems were identified as a top15-diagnosis, we did not include it as an outcome because it is based on a list of biochemical input-data with simple limits, in which case physicians require no assistance from machine learning. Consequently, we ended up with 19 outcomes (14 diagnosis-based and 5 non-diagnosis-based).

#### Nurse data registrations and sampling

When the patient is admitted the nurse performs triage and registers clinical and personal data for all patients. Critical score (Aggregate weighted scoring system: TOKS)^15^ is performed on indication based on the triaging clinical score. Some data are registered based on urine availability or on a select patient group as height and weight. This leads to low data availability for urine samples and height and weight. We therefore chose to exclude height, weight and body mass index from further analysis. Urine dipstick testing was performed by nurses and the data registered electronically. All data registered by nurses and used for this project were extracted electronically (see Table S1).

#### ECG

All patients had a 12-lead resting ECG performed using the MAC 5500 (GE Healthcare, Chicago, IL, USA) at admission. A total of 26 ECG features (see suppl. Table S1) were extracted from each ECG using the ECG Muse software (GE HealthCare, Chicago, IL, USA).

#### Statistics

To test whether performing more analyses early on would improve the treatment logistics we investigated the average number of extra venipunctures in the 24 h following admission. This was done in Vejle hospital only, since all suspected Covid19-cases were admitted to Kolding Hospital. Covid19 leads to an increased number of isolated patients in which case the patients may have extra analyses performed in the initial venipuncture and hence clouding the investigation. The proportion of extra venipunctures in the study period compared to before and after was performed using Chi squared test. All other categorical data was analyzed using chi squared test. Continuous data were analyzed using Mann–Whitney U-test. All statistics were performed using Graphpad Prism 9.2.0 (Graphpad Software, Boston, MA, USA) or SAS VIYA V.03.05 (SAS Institute, Cary, NC, USA).

Sensitivity analyses were performed on urine analyses since these have low data availability (high degree of missing).

### Machine learning

All input variables were grouped into a list of feature packages as shown in Table 1 in order to improve the data analysis flow. Only data available when the biomarkers are analyzed approximately 60 min after admission are used as input features for training algorithms. All included patients were randomly divided into a training dataset (80%) and a holdout dataset (20%), this method was chosen to get comparable cohorts and avoid seasonal changes in the diseases. The training set was only used for training and the holdout set was only used afterwards to estimate reliability in performances. Model training was performed using tenfold cross validation without replacement with each fold successively used as holdout used for hyperparameter optimization and error estimation with AUC as the primary criterium. For each target, we performed training using Random Forest, Gradient Boosting and logistic regression models. Neural network models were applied to four selected outcomes in order to ascertain the possible advantages of this classifier. Since Random Forest and Gradient Boosting models were always superior to neural networks when comparing AUC, we did not apply neural networks to other outcomes. Since regression and neural networks require non-missing dataset, missing data was imputed for these analysis methods. It was important for further studies to use a method that could be used for variable-reduction across multiple algorithms: Variables describing number of previous diagnoses are imputed with 0. Variables describing number of days since a previous diagnosis are imputed with 1,825 (5 years) since this was the maximum possible time for historical diagnoses. All other continuous variables were imputed using the median from the entire training set. Categorical variables were imputed with a missing class ‘-99’. Regression (forward selection) and neural networks were trained using either all variables or all blood parameters together with age and gender. Cross validation AUC was used as the main criterium for algorithm selection. Random Forest and Gradient Boosting were trained initially using the blood variables package only performing grid search with the parameters trees = 25, 50, 75, 100; number of bins = 30, 50, 100; and fraction = 0.5, 0.6, 0.7 for Random Forest and trees = 20, 40, 60; sampling rate = 0.5, 0.6, 0.7; number of bins = 30, 50; and learning rate = 0.05, 0.1. The best performing model for each classifier was then trained using all feature packages in a forward selection method in order to include all features in the selection process. The final step was to optimize hyperparameters using the best performing feature packages. As the final models had been trained, the models were applied to the holdout dataset for the true generalization performance.

For each algorithm the AUC on the Receiver Operating Characteristics curve (ROC) was calculated in both the training and holdout cohorts. To compare algorithms in an objective and non-biased way we identified cutoff-points for each algorithm for maximized sensitivity and specificity using Youden’s Index in the training cohort and calculated relevant measures of accuracy F1-score, sensitivity (recall), specificity and positive predictive value (precision) from this cutoff in the holdout cohort. This leads to more uniform sensitivity and specificity which is often not wanted in the emergency patients with low prevalence of each disease, but was chosen in order to report objectively and to make it possible to compare algorithms. For each algorithm, we produced a calibration plot by using the cutoff values by splitting the training dataset into 10 sets of equal number of patients and applying the cut-off values on the holdout dataset.

### Ethics

The Helsinki Declaration was followed and fulfilled. Data protection was conducted according to EU GDPR rules, as no data left the Region of Southern Denmark’s firewall and log-in protection domain and a DPIA (Data Protection Impact Assessment) was completed.

The study was performed as a retrospective registry-study. Permission to perform this study was issued by Health Authorities of the Region of Southern Denmark, journal nr. 18/62512. Informed consent was waived under the Danish Health Law, paragraph 42, section 2. All patients were informed on the new procedure of increasing the number of analyses for diagnostic purposes, all patients accepted. The total volume of blood for analysis was increased to 38 mL for each patient at the admittance at is normally the case for 22% of all patients. This volume does not constitute a physiological problem in adults.

## Results

A total of 9190 patient admissions were included in this study. All patients underwent the same standard blood tests, further blood tests could be requested based on clinical needs and urine samples were taken based on clinical decisions.

**Figure 1:**
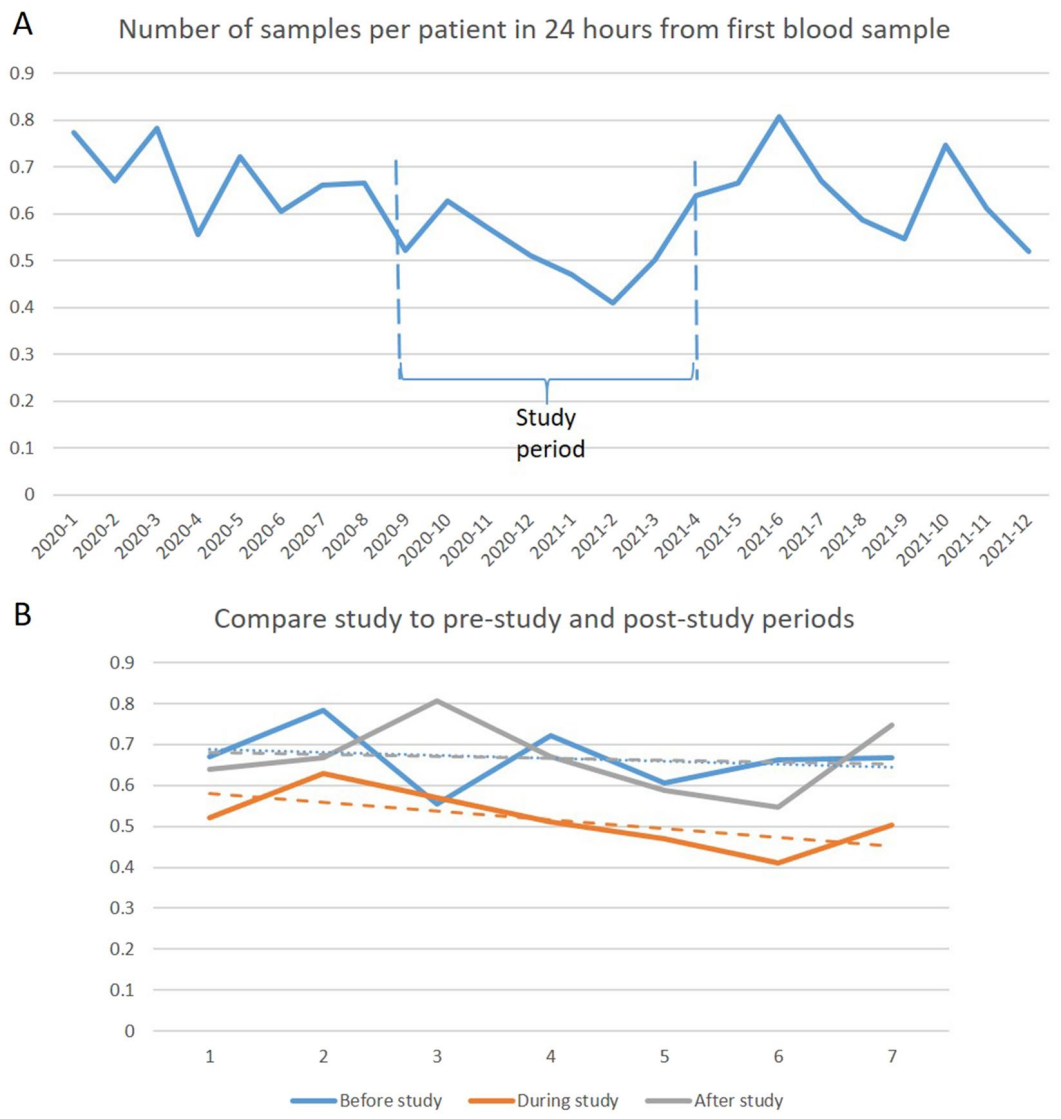
(**A**) Blood sampling impact. The average number of venipuncture samples in the 24 h following the Desert-sample as a function of time(year-month) in Vejle Hospital was reduced by 22% in the study period (p = 0.00002). (**B**) Prestudy, study and post-study 7 months compared with regression lines of same color.

The average number of new blood venipunctures in the 24 h following the admission venipuncture was reduced by 22.3% from an average of 0.664 before and after the study period to 0.516 per patient during the study period (p = 0.00002) which equals to an odds ratio of 0.55(95% CI 0.48–0.63) as seen in Fig. 1.

All biomarker reports had a high data availability as can be seen in Table 1 and Table S1. All patients had blood drawn. The data availability of measures performed by the nurse were also good (96.8–99.2%) except for weight and height (66.7–67.1%). The data availability for sampling urine laboratory analyses was (37.3–39.1%) due to a decision of sampling only on clinical indication. The availability rates for sampling for point-of-care-testing-measures were in the interval (40.6–55.3%).

**Figure 2:**
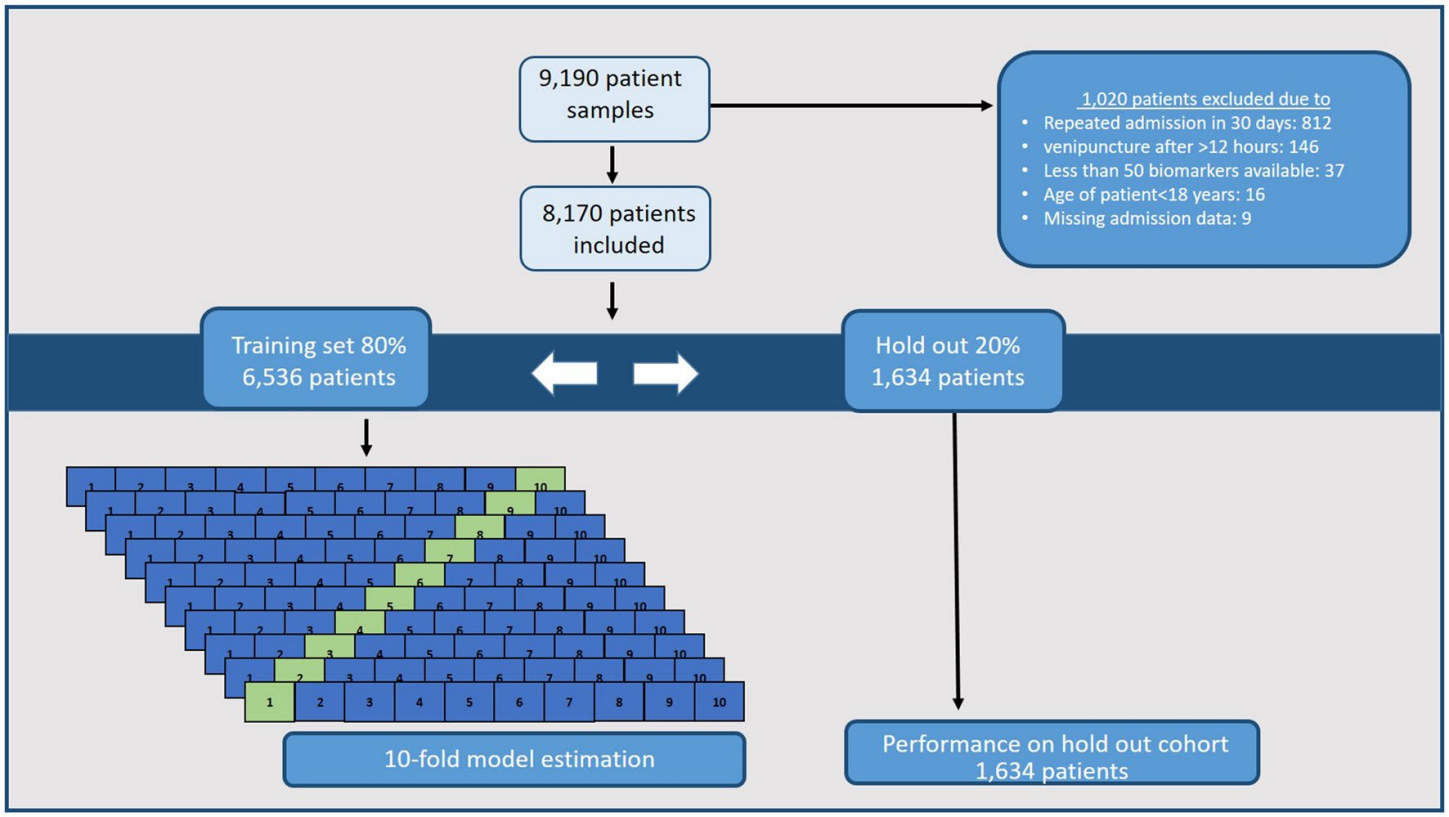
Flow—developing machine learning models showing first the exclusion criteria, then the 80/20% split for training and holdout cohorts. The training cohort was used to train all models using tenfold cross validation. Finally the 20% holdout cohort was used to investigate the performance of each algorithm on a dataset not used previously for training, selection or hyperparameter optimization.

The two cohorts for training and holdout validation were randomly divided as shown in Fig. 2. There were no difference in the basic characteristics between the two cohorts as detailed in Table 2. We also tested for difference between the hospital-subgroups and found significant differences between the two hospitals for which Kolding Hospital had higher Charlson Index in both training (Kolding 0[0–2] (median[IQR]) vs. Vejle 0[0–2], p = 0.0001) and holdout cohorts (Kolding 0[0–2] (median[IQR]) vs. Vejle 0[0–1], p = 0.02). Kolding Hospital also had a significantly higher mortality rate within 7 days (3.0% vs. 2.1%; p = 0.02) and higher readmission rate (12.4% vs. 10.1%; p = 0.003).

**Table 2:** Patient characteristics between the training and holdout cohorts.

Variable	Total	Training cohort	Holdout cohort	Difference between cohorts, p-value
N	9190	6536	1634	N/A
Sex, % male	50.9%	50.4%	51.8%	0.32
Age, median [IQR]	68.1 [50.7–80.0]	67.9 [50.3–79.7]	67.4 [49.7–79.8]	0.89
Charlson, median [IQR]	0 [0–2]	0 [0–2]	0 [0–2]	0.24
Die within 7 days, %	2.6%	2.6%	2.6%	0.94
Die within 30 days, %	6.4%	5.8%	6.7%	0.18
Readmission, %	14.1%	13.1%	12.8%	0.74

All algorithms trained are listed in Table 3 as well as their performances in terms of F1-score, sensitivity (recall), specificity and positive predictive value (precision) in the training cohort as well as the validated AUC in the holdout cohort. All algorithms obtained areas under the ROC-curves in the range of 71.8–96.3% in the holdout cohort and 68.3–98.2% in the training cohort. Examples are death in days (AUC 91.4%), death in 30 days (AUC 91.3%), sepsis (AUC 90.1%) and safe-discharge (AUC 87.3%).

**Table 3:** Model results—For each target outcome is shown AUC for the training cohort and thereafter AUC, Recall, specificity, precision, negative predictive value, F1-score and accuracy for each model on the holdout cohort based on a cut-off threshold determined by Youden’s Index.

Outcome	Training cohort		Holdout cohort							
	Number of patients with outcome	AUC (%)	Number of patients with outcome	AUC	Sensitivity (recall)	Specificity	Precision (Positive predictive value)	Negative predictive value	F1 score	Accuracy
Chronic pulmonary disease	606	92.3	149	89.9% [87.0–92.6]	77.2% [70.4–83.9]	86.1% [84.3–88.0]	35.8% [31.0–41.0]	97.4% [96.5–98.2]	48.9% [43.5–54.1]	85.3% [83.5–87.1]
Acute alcohol consumption	108	98.2	20	96.3% [90.4–99.4]	95.0% [83.3–100]	93.9% [92.7–95.1]	16.2% [9.7–23.7]	99.9% [99.8–100]	27.7% [17.5–38.0]	93.9% [92.7–95.0]
Chronic alcohol consumption	340	97.2	72	95.3% [91.8–98.1]	90.3% [82.4–96.8]	91.8% [90.5–93.1]	33.7% [27.0–40.8]	99.5% [99.1–99.9]	49.1% [41.2–56.5]	91.7% [90.4–93.0]
Urinary tract infection (UTI)	319	84.0	49	85.3% [79.8–89.8]	85.7% [75.5–94.9]	73.1% [70.9–75.3]	9.0% [6.6–11.8]	99.4% [98.9–99.8]	16.2% [12.2–20.7]	73.5% [71.3–75.7]
Pneumonia	414	86.6	102	85.9% [82.6–88.9]	84.3% [76.7–90.9]	70.4% [68.1–72.7]	15.9% [12.8–18.9]	98.5% [97.7–99.2]	26.8% [22.1–31.1]	71.2% [69.0–73.5]
Erysipelas	108	88.5	35	87.3% [81.9–91.8]	62.9% [45.3–78.1]	88.5% [86.9–90.1]	10.7% [6.4–15.3]	99.1% [98.6–99.5]	18.3% [11.3–25.2]	87.9% [86.3–89.5]
Sepsis	154	90.1	45	90.0% [82.7–95.5]	83.9% [71.0–96.2]	85.5% [83.8–87.3]	10.1% [6.7–13.9]	99.6% [99.3–99.9]	18.0% [12.3–23.9]	85.5% [83.8–87.3]
Other infection	100	75.3	25	74.5% [63.7–84.2]	72.0% [52.0–88.7]	71.8% [69.4–74.0]	3.8% [2.2–5.6]	99.4% [98.9–99.8]	7.2% [4.3–10.5]	71.8% [69.5–74.0]
Intoxication	139	84.1	33	82.5% [74.4–89.0]	78.8% [64.3–91.9]	71.0% [68.9–73.2]	5.3% [3.5–7.3]	99.4% [98.9–99.8]	9.9% [6.6–13.5]	71.2% [69.0–73.3]
Gastroenterology	208	68.3	58	71.8% [65.5–78.0]	81.0% [70.7–90.3]	51.3% [48.7–53.6]	5.8% [4.3–7.5]	98.7% [97.8–99.4]	10.8% [8.1–13.7]	52.3% [49.9–54.7]
Chest pain	365	92.1	75	92.2% [89.2–94.7]	86.7% [78.7–93.9]	82.0% [80.1–83.9]	18.8% [14.7–22.9]	99.2% [98.7–99.7]	30.9% [25.1–36.4]	82.2% [80.3–84.0]
Kidney insufficiency	140	97.2	30	92.5% [85.5–98.0]	83.3% [68.4–96.4]	92.4% [91.0–93.7]	17.0% [11.0–23.7]	99.7% [99.3–99.9]	28.2% [19.3–37.2]	92.2% [90.8–93.5]
Syncope and malaise	329	79.6	92	77.9% [73.6–82.5]	66.3% [56.6–76.1]	71.9% [69.6–74.1]	12.3% [9.6–15.2]	97.3% [96.3–98.2]	20.8% [16.5–25.0]	71.6% [69.4–73.7]
COVID19	225	94.4	65	93.1% [89.1–96.4]	80.0% [69.9–89.4]	91.0% [89.4–92.4]	26.9% [21.0–33.3]	99.1% [98.6–99.6]	40.3% [32.7–47.6]	90.6% [89.0–91.9]
Die within 7 days	170	91.4	43	92.5% [89.6–95.0]	76.7% [63.2–88.0]	87.1% [85.2–88.7]	13.8% [9.6–18.6]	99.3% [98.8–99.7]	23.4% [16.7–30.3]	86.8% [85.0–88.5]
Die within 30 days	379	91.3	109	89.2% [86.3–91.7]	89.0% [82.5–94.5]	75.7% [73.5–77.9]	20.7% [17.1–24.4]	99.0% [98.4–99.5]	33.6% [28.5–38.5]	76.6% [74.4–78.6]
Repirator treatment	81	89.5	36	90.8% [83.9–96.3]	88.2% [76.7–97.5]	79.1% [77.0–81.0]	8.2% [5.7–11.1]	99.7% [99.4–99.9]	15.0% [10.7–19.7]	79.3% [77.2–81.2]
Intensive care stay	100	90.9	34	90.6% [84.7–95.3]	86.1% [74.7–96.8]	87.1% [85.5–88.9]	13.1% [9.0–17.5]	99.6% [99.3–99.9]	22.7% [16.2–29.3]	87.1% [85.4–88.9]
Safe-discharge	2893	87.3	731	89.5% [88.0–91.0]	82.9% [80.1–85.6]	79.6% [77.1–82.1]	76.7% [73.7–79.7]	85.2% [82.9–87.6]	79.7% [77.3–81.9]	81.1% [79.3–83.0]

**Figure 3:**
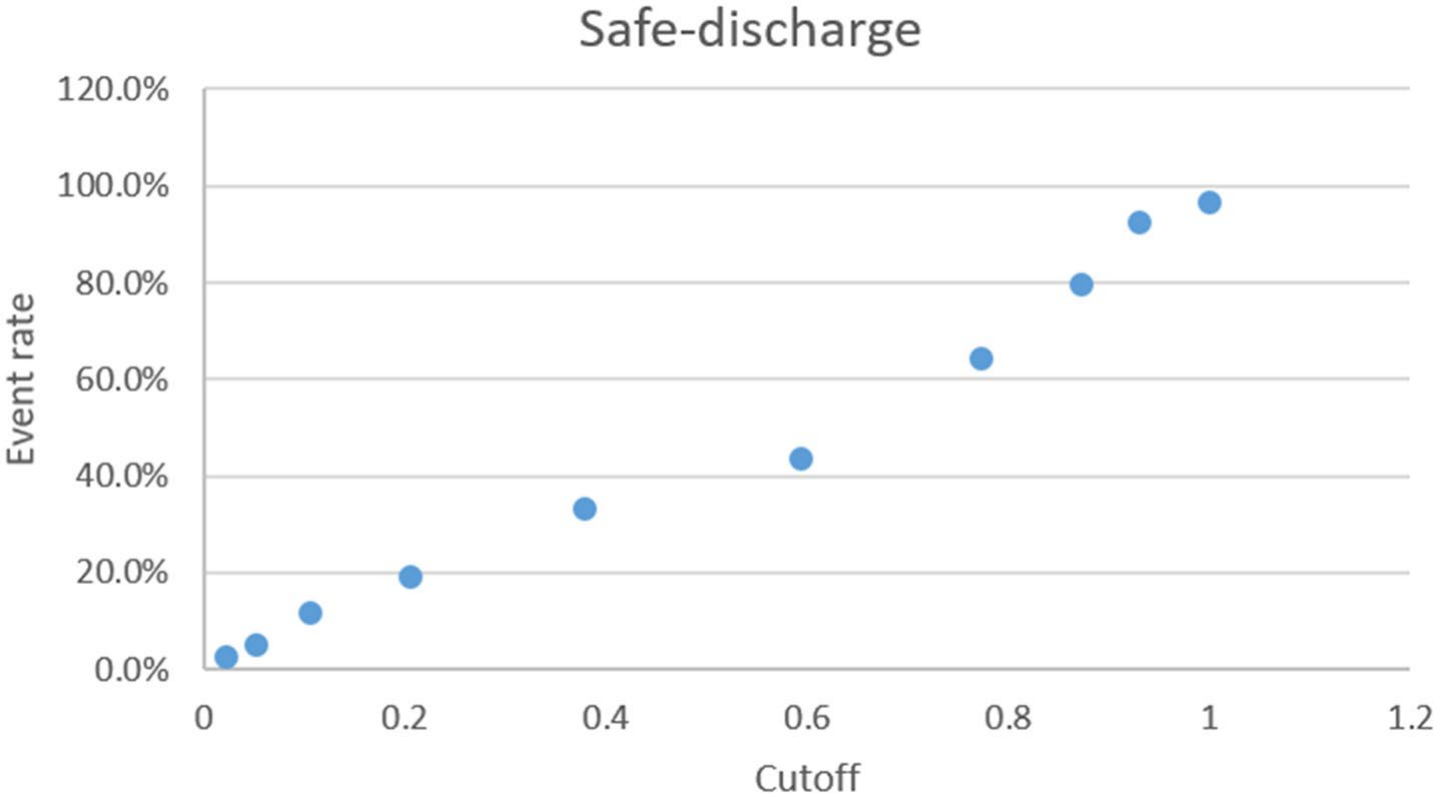
A calibration plot of the algorithm for safe discharge showing the risk for each of the 10 subdivisions of the holdout cohort. Cutoffs are determined by the training cohort.

Calibration plots for the safe discharge algorithm is depicted in Fig. 3 and calibration plots for all algorithms can be found in supplemental Figs. S1, S2, S3, S4, S5. It is seen that the algorithm for other infection does not have a continuously increasing risk as the cut-off limits increase.

Panic limits were exceeded in a number of patients as listed in Table S3. Especially fibrin d-dimer, creatinine kinase and troponine t were reported frequently.

## Discussion

Using biomarker data from 9190 patient admissions at the medical emergency departments of two hospitals, we were able to train and validate 19 machine learning algorithms that exhibited sufficiently high performance for use as a statistical support tool in a clinical setting. These algorithms were validated in a separate holdout cohort similar to the training cohort. Both cohorts were randomly allocated.

By the use of this standard repertoire we observed a significant reduction, averaging 22.3%, in new venipunctures within 24 h of the admission venipuncture. This is of clinical importance since it is difficult to reduce the number of venipunctures in the clinical setting without disrupting the diagnostic or monitoring process. This shows that increasing the number of analyses performed in the early phase of admission improves patient treatment logistics and possibly accelerate diagnostics. Of course, there are many analyses, such as repeating abnormal results, which necessitates new venipunctures and are challenging to eliminate.

The list of biomarkers consist of high quality laboratory biomarkers although the urine analyses had low availability rate due to sampling only on clinical request. Other variables ranged from very high data availability to fairly low, e.g. height and weight. Due to this and risk of bias, height and weight were not used as input data in the algorithm training.

Patients were randomly allocated to either the training or holdout cohorts, and we found no significant difference between them on any metric. We did however find significant difference between the two hospitals, which is not surprising, since Kolding Hospital is the major emergency hospital of the two receiving more severe cases. This difference was clear with higher Charlson Index, higher mortality within 7 days and a higher rate of readmission within 30 days. These differences are expected and should not impact the training and validation of the algorithms.

By including many biomarkers which are realistic for hospital laboratories to deliver within 60–80 min, we have shown that it is possible to train a list of algorithms for a variety of outcomes seen in a medical emergency department. For some target outcomes the algorithms show great promise, while other exhibit more modest predictive capabilities as evidenced by their respective AUC values. However, determining the practical utility of each algorithm for physicians remains challenging. Logically, good performance would equal high usability, but without comparing physicians without algorithm support to physicians with algorithm support, we do not know whether each algorithm or the total number of algorithms actually helps the physician or not. Algorithms such as death in 30 days, sepsis and safe-discharge support outcomes difficult to predict and it seems that we have included the majority of input variables important for these outcomes. In the case of gastroenterology it seems that we have not included the important variables obtaining a low AUC. We did not include data on patient symptoms and since symptoms such as abdominal pain, vomiting and diarrhea are available to the physician we do not believe that this algorithm will prove helpful. The algorithm for other infection is covering all other infections besides the ones that constitute separate algorithm outcomes. The performance is not that good and it is probably an example of a poor ensample outcome. The alternative would have been all infections as one, but clinically the physicians probably do not need help identifying infections as a whole except for certain specific cases. In this study we chose an objectively determined cut-off for each algorithm in order to compare all algorithms. To determine the future use of each algorithm it would either be necessary to treat the results as dichotomous by choosing a cut-off threshold for each algorithm based on the precise situation of use optimizing either sensitivity or specificity. Another solution is to translate each model result into absolute risk, this is, however, not the typical way results are presented to the clinicians and would hence require training. The algorithms could be used as diagnostic support or as a second opinion, especially in situations with high patient load, stressed personal or during the night, late in a shift, unexperienced clinicians etc. In our hospitals, the clinicians see the patient as soon as possible following triage and then each patient is revisited within approximately 4 h from admission. The algorithms could then be used either at first or second time the clinician sees each patient. Using percentage risk for each disease/condition for each patient makes it possible to diagnose more precisely and objectively as well as making communication between physicians easier and possibly more detailed. It is very important that the physicians view algorithms as tools and learn how and when to use them^16^. Whether this would be easier with dichotomous or quantitatively algorithm results remains to be studied. In this study we only performed one venipuncture and hence can only train algorithms at admission. Some of the algorithms may however also be used to monitor patients for treatment response, e.g. in sepsis.

Other researchers have developed algorithms somewhat comparable to those developed in this project. Soltan et al. developed a COVID-19 algorithm based on laboratory data and vital parameters which can be available within one hour from patient arrival to hospital with a performance of AUC 88.1% compared to our COVID-19 algorithm at AUC 93.1% both in validation cohorts^17^. Machine learning algorithms for identifying sepsis in the ED have been developed by Lin et a. with an AUC of 75% in external validation cohort outperforming qSOFA^18^. Our developed algorithm for sepsis has an AUC of 90.0% in the validation cohort. It is, however, difficult to compare studies in sepsis due to several definitions of the disease. Our algorithms for mortaily for both 7 and 30 days outperformed the SERP + algorithms^10^.

Although AUC for the algorithms may be close to 100% we still see modest positive predictive values due to low prevalence of several diseases in the cohorts. This is, however, also the case for the other tools physician use^19^. To investigate whether an algorithm is beneficial to physicians in a clinical setting a randomized controlled trial should be performed. This would be no easy task since the difference in outcome, e.g. 30 day mortality, in the two groups would be limited and thus requiring a large sample size in order to reach significance.

Increasing the use of biomarkers may lead to an increase in expenses. Transitioning from the development phase to application, the number of biomarkers needed will be markedly reduced. Also, the expenses for the initial blood workup is limited compared to the total hospital cost, so increasing the initial expenses early on is expected to reduce the total cost of the hospital stay.

One might argue for an ethical issue in ordering biomarkers without having a physician signing off on all results. There are reasons why all biomarkers are not normally analyzed for all patients, one being financial costs another being the risk of increasing the number of false positive results. However, choosing not to use algorithms due to these arguments would lead to stagnation. Having physicians look through all results would hinder the potentially positive effects of the algorithms. We handled this instead by using panic values.

Panic limits were triggered in a significant number of patients. Locally, there should be an ongoing discussion about which biomarkers should have panic limits and the threshold at which they should be set. Set too high, important information may be overlooked, set too low the physicians are alerted for unimportant cases. In this case, results were produced but not automatically delivered to the physicians thus making appropriate panic values more important than normally. Further research in this area is necessary as we believe the use of algorithms will reduce the importance of reference limits when the actual biomarkers quantitative value is used instead.

## Limits and strengths

We present a study here with a vastly expanded standard list of biomarkers used to train algorithms for predicting each target making it possible to reach high AUC’s and expand the range of biomarkers showing predictive value. We included a large number of patients from two hospitals. All patients had the exact same list of blood biomarkers performed removing physician selection as a bias. All patients were included consecutively on both hospitals aside from a 13-day Christmas break. All biomarkers undergo quality control multiple times daily as is the standard in accredited hospital laboratories.

There are also some limitations to this study. Although all patients had the same blood biomarkers analyzed, urine was analyzed only on clinical indication. We used the diagnoses coded by the physicians during the entire admittance and in some cases the 30 days following the admittance. When diagnosing without using the gold standard for diagnostics there is a risk for misdiagnosing and thereby training the algorithms on a wrong outcome, hence resulting in an incorrect AUC. Although we included a large amount of patients, some target outcomes were infrequent making it difficult/impossible to investigate subgroups. Patients were included over a period of approximately 7 months making seasonal changes in diseases a source of risk for overfitting models. The study period started approximately 6 month following covid19 pandemic hit Denmark. This made covid19 prevalent during this period although it might not be an issue later on. The clinical departments change their settings from time to time in order to improve logistics. Hence, algorithms trained in one period may deteriorate over time. We used 20% holdout sample for all algorithms, which may suit very prevalent diseases better than not so prevalent diseases. Due to different levels for biomarkers according to racial differences it is a limitation that we did this study on a Danish population which is primarily Caucasian.

## Conclusion

We have developed 19 machine learning algorithms for use in predicting which disease or generalized outcome will happen for medical emergency patients in two Danish hospitals. We have validated the results in a holdout cohort. Further prospective validating of the results and algorithms is needed in order to ascertain whether these tools will be valuable to physicians in the clinical setting. In an emergency setting, where a patient’s health can deteriorate rapidly, the time between patient admission and the availability of algorithm results is crucial. This requires a quick blood draw, fast transport to the laboratory and automated equipment together with efficient dataflow. In case any of these factors break down the algorithms arrive at the physicians later with less possible impact.

Although these algorithms were developed using a single point-in-time sample, several of these tools could also possibly prove helpful in monitoring disease, e.g. the death- and sepsis algorithms. The covid-19 algorithm is probably not specific to covid-19 but could be a general tool for virus-infection or possibly virus infections in the upper-respiratory tract. Future studies should focus on validating our findings both in other hospital setting, with different laboratory instruments producing equivalent analysis results, in other cohort with other races. Prospective validating in the hands of the clinicians is very important. Increasing the number of algorithms tested for each outcome may increase the performance. Investigating whether clinicians prefer algorithm results as dichotomous or quantitative values is a very important subject in order to validate prospectively. Further studies on which analyses result in reducing further venipunctures within 24 h is an important research area.

### Supplementary Information

Supplementary Legends.

Supplementary Figure S1.

Supplementary Figure S2.

Supplementary Figure S3.

Supplementary Figure S4.

Supplementary Figure S5.

Supplementary Tables.

## Data Availability

The datasets generated during and/or analysed during the current study are not publicly available due to legal and privacy issues but are available from the corresponding author on reasonable request.

## Abbreviations

ROC: Receiver operating charateristics
UTI: Urinary tract infection
ECG: Electrocardiogram
ABL: Acid base laboratory
DPIA: Data protection impact assessment
AUC: Area under the curve
ED: Emergency department
