# Supplementary material for: Machine learning in diagnostic support in medical emergency departments
Source: Sci Rep. 2024 Aug 2;14:17889. doi: 10.1038/s41598-024-66837-w (PMC11297196; doi:10.1038/s41598-024-66837-w)
Supplement: Supplementary file 1 — Supplementary Legends. [file 41598_2024_66837_MOESM1_ESM.docx]

Legend for figure s1-s5:

Calibration plots for all the algorithms developed in this study showing the risk for each of the 10 subdivisions of the holdout cohort. Cutoffs are determined by the training cohort.
