# Supplementary figures and images for: Machine learning in diagnostic support in medical emergency departments
Source: Sci Rep. 2024 Aug 2;14:17889. doi: 10.1038/s41598-024-66837-w (PMC11297196; doi:10.1038/s41598-024-66837-w)

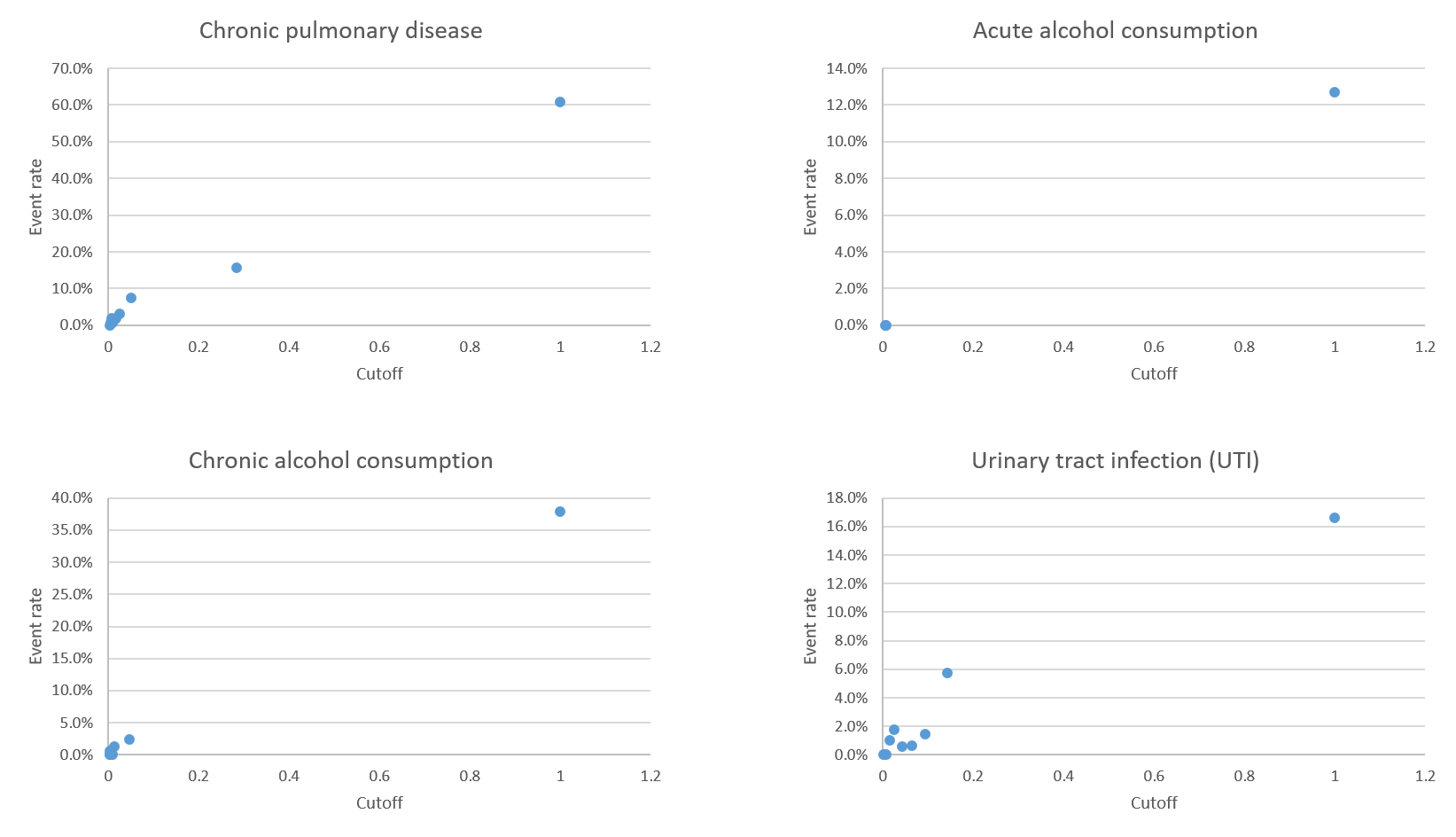

Supplement: Supplementary file 2 — Supplementary Figure S1. [file 41598_2024_66837_MOESM2_ESM.tif]

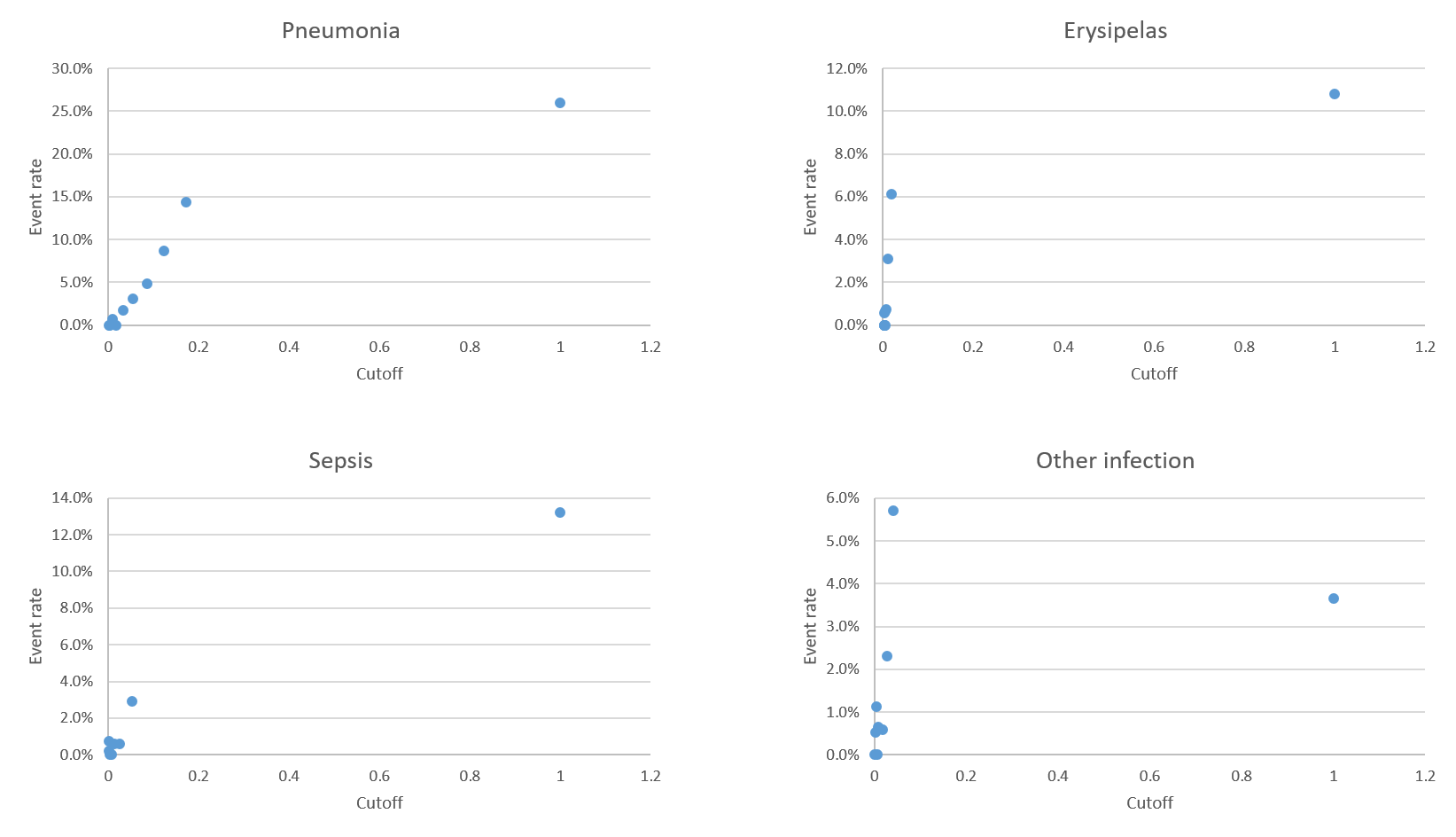

Supplement: Supplementary file 3 — Supplementary Figure S2. [file 41598_2024_66837_MOESM3_ESM.tif]

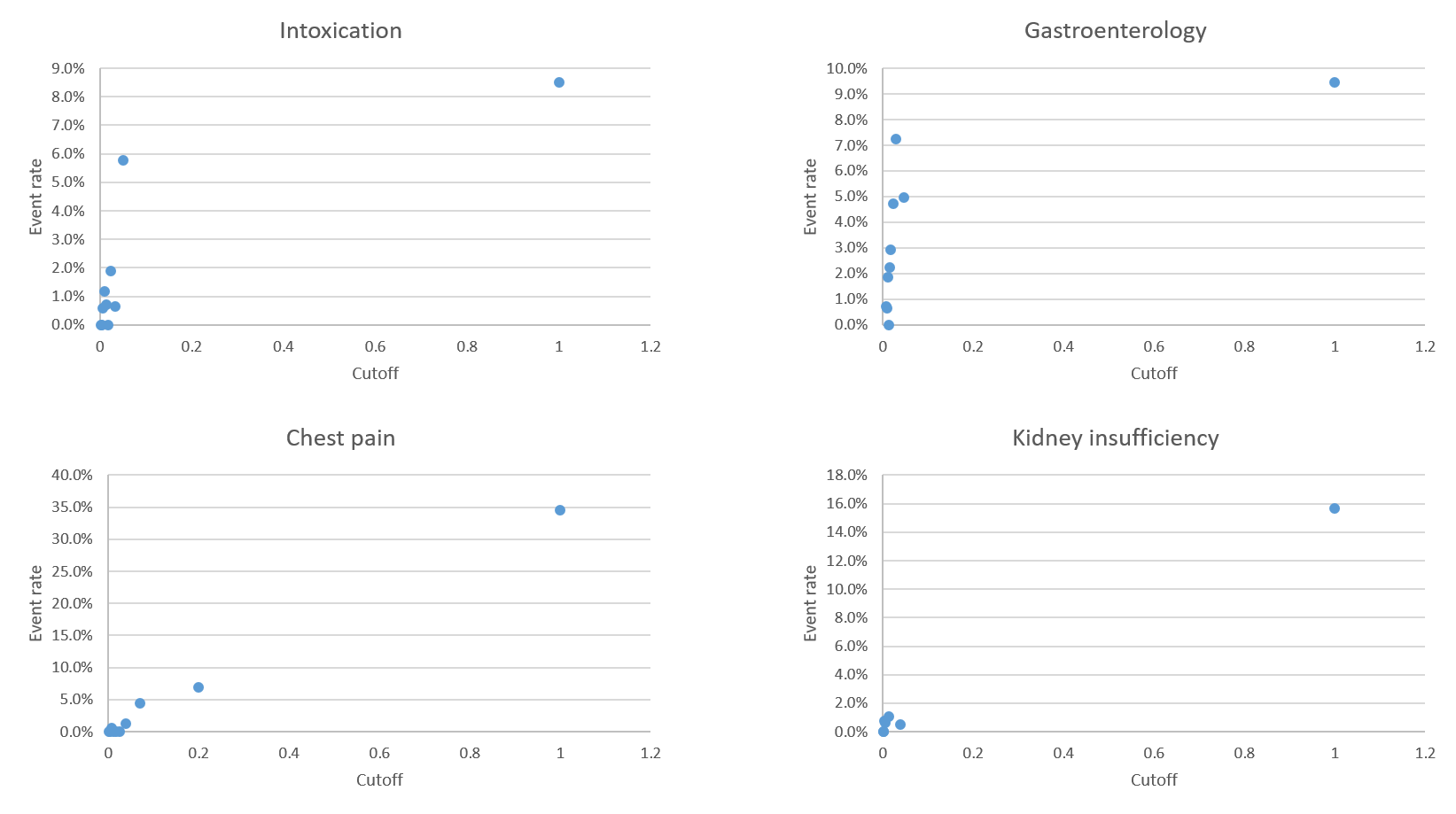

Supplement: Supplementary file 4 — Supplementary Figure S3. [file 41598_2024_66837_MOESM4_ESM.tif]

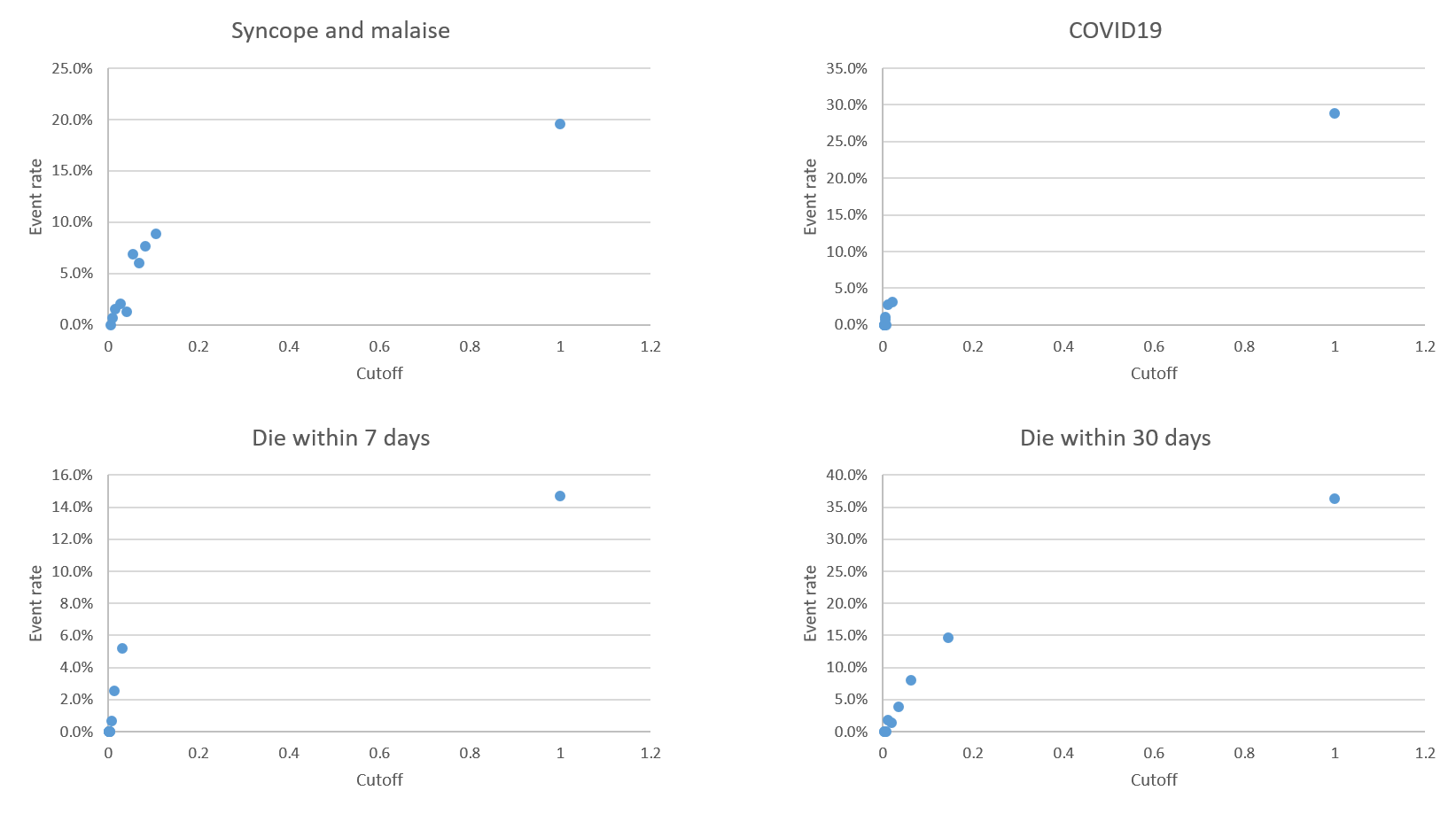

Supplement: Supplementary file 5 — Supplementary Figure S4. [file 41598_2024_66837_MOESM5_ESM.tif]

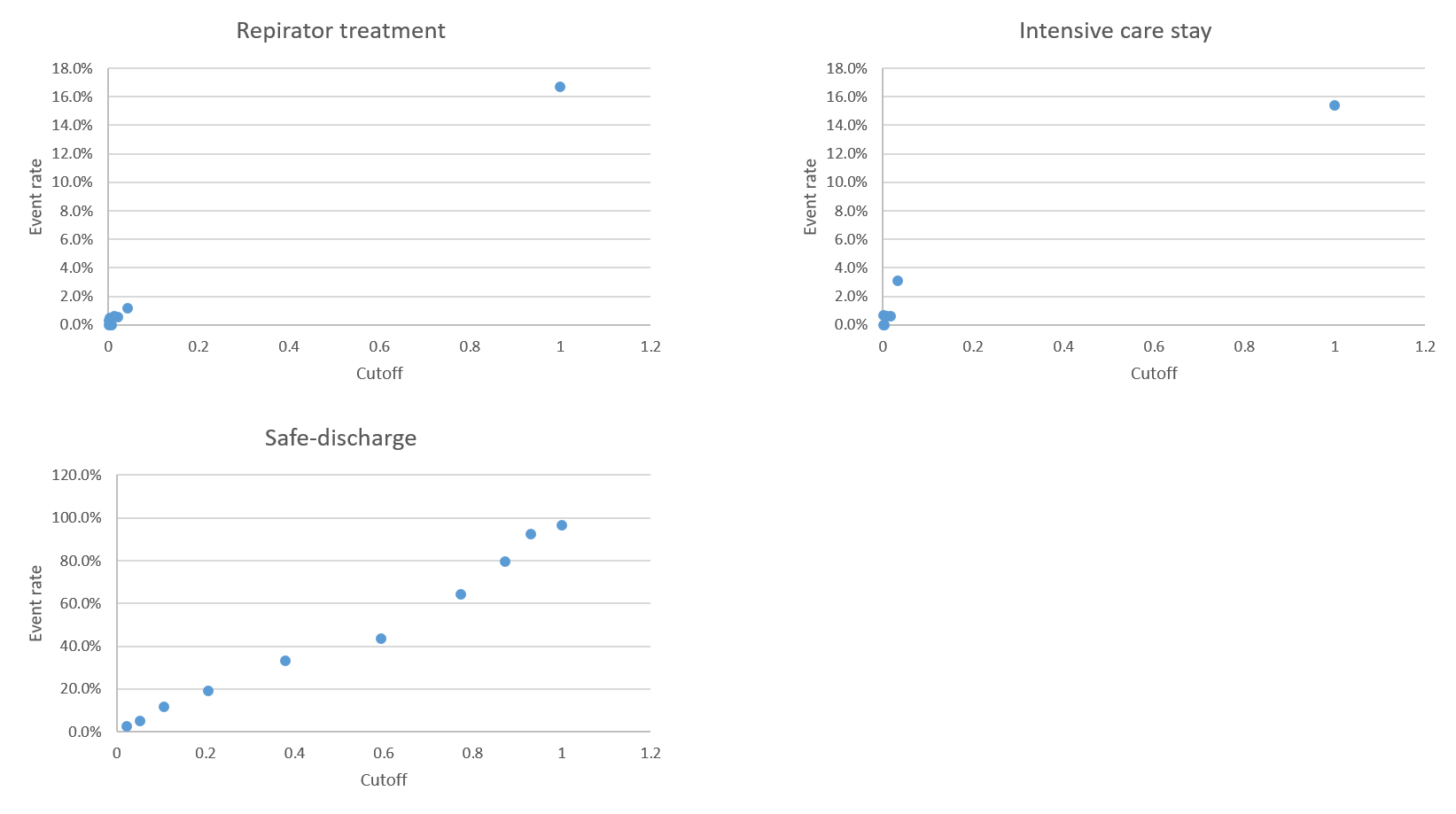

Supplement: Supplementary file 6 — Supplementary Figure S5. [file 41598_2024_66837_MOESM6_ESM.tif]
