# Supplementary material for: Machine learning in diagnostic support in medical emergency departments
Source: Sci Rep. 2024 Aug 2;14:17889. doi: 10.1038/s41598-024-66837-w (PMC11297196; doi:10.1038/s41598-024-66837-w)
Supplement: Supplementary file 7 — Supplementary Tables. [file 41598_2024_66837_MOESM7_ESM.docx]

Supplemental table S1

Data sources and features. For each variable, the data availability is listed.

| VariablePackage | Input name | Data availability,% | Data availability |
| --- | --- | --- | --- |
| Blood(gas tension and acid/base, correlated) | Actual base excess of fully oxygenated blood | 96.0% | 8822/9190 |
| Blood(gas tension and acid/base, correlated) | Standard base excess | 96.2% | 8838/9190 |
| Blood(gas tension and acid/base, correlated) | Standard base excess of fully oxygenated blood | 96.0% | 8822/9190 |
| Blood(gas tension and acid/base, correlated) | Hydrogen ion concentration | 96.2% | 8839/9190 |
| Blood(gas tension and acid/base, correlated) | O2 capacity | 96.1% | 8830/9190 |
| Blood(gas tension and acid/base, correlated) | Standard pH | 96.0% | 8822/9190 |
| Blood(gas tension and acid/base, correlated) | Osmolality | 96.1% | 8833/9190 |
| Blood(gas tension and acid/base, correlated) | total CO2 in plasma | 96.2% | 8838/9190 |
| Blood(gas tension and acid/base, correlated) | total oxygen | 96.0% | 8824/9190 |
| Blood(gas tension and acid/base, correlated) | Hydrogenbicarbonate | 96.2% | 8838/9190 |
| Blood(hematology, research) | Basophil count based on WDF channel | 97.7% | 8980/9190 |
| Blood(hematology, research) | Basophil percent based on WDF channel | 97.7% | 8981/9190 |
| Blood(hematology, research) | Basophil percent based on WNR channel | 97.7% | 8981/9190 |
| Blood(hematology, research) | Difference of hemoglobin concentration between HGB and HGB-O | 8.2% | 751/9190 |
| Blood(hematology, research) | Fractional Reticulocytes | 97.5% | 8957/9190 |
| Blood(hematology, research) | Fractional Reticulocytes, % | 97.5% | 8960/9190 |
| Blood(hematology, research) | Lymfocytes, upper area | 97.7% | 8981/9190 |
| Blood(hematology, research) | Lymfocytes, upper area, % | 97.7% | 8980/9190 |
| Blood(hematology, research) | Platelet fraction, stronger intensity, IPF | 6.0% | 548/9190 |
| Blood(hematology, research) | Platelet intensity, forward, IRF | 97.7% | 8981/9190 |
| Blood(hematology, research) | Lymphocytes, % | 97.7% | 8978/9190 |
| Blood(hematology, research) | Lymphocytes, lateral distribution width, WDF | 97.7% | 8981/9190 |
| Blood(hematology, research) | Lymphocytes, fluorescent light distribution width, WDF | 97.7% | 8981/9190 |
| Blood(hematology, research) | Lymphocytes, forward distribution width, WDF | 97.7% | 8981/9190 |
| Blood(hematology, research) | Lymphocytes, lateral intensity, WDF | 97.7% | 8981/9190 |
| Blood(hematology, research) | Lymphocytes, fluorescent intensity, WDF | 97.7% | 8981/9190 |
| Blood(hematology, research) | Lymphocytes, forward intensity, WDF | 97.7% | 8981/9190 |
| Blood(hematology, research) | Monocytes, lateral distribution width, WDF | 97.7% | 8981/9190 |
| Blood(hematology, research) | Monocytes, fluorescent light distribution width, WDF | 97.7% | 8981/9190 |
| Blood(hematology, research) | Monocytes, forward distribution width, WDF | 97.7% | 8981/9190 |
| Blood(hematology, research) | Monocytes, lateral intensity, WDF | 97.7% | 8981/9190 |
| Blood(hematology, research) | Monocytes, fluorescent intensity, WDF | 97.7% | 8981/9190 |
| Blood(hematology, research) | Monocytes, forward intensity, WDF | 97.7% | 8981/9190 |
| Blood(hematology, research) | Neutrophilocytes, forward intensity, WDF | 97.7% | 8979/9190 |
| Blood(hematology, research) | Neutrophilocytes, fluorescent intensity, WDF | 97.7% | 8978/9190 |
| Blood(hematology, research) | Neutrophilocytes, lateral intensity, WDF | 97.7% | 8980/9190 |
| Blood(hematology, research) | Neutrophilocytes, lateral distribution width, WDF | 97.7% | 8981/9190 |
| Blood(hematology, research) | Neutrophilocytes, fluorescent light distribution width, WDF | 97.7% | 8981/9190 |
| Blood(hematology, research) | Neutrophilocytes, forward distribution width, WDF | 97.7% | 8981/9190 |
| Blood(hematology, research) | Reticulocyte count in UPP area | 97.7% | 8981/9190 |
| Blood(hematology, research) | Reticulocytes, forward intensity, RET scattergram | 97.7% | 8981/9190 |
| Blood(hematology, research) | Ratio of immature granulocyte to Leucocytes, % | 97.7% | 8975/9190 |
| Blood(hematology, research) | Immature platelet fraction | 6.0% | 548/9190 |
| Blood(hematology, research) | Immature platelet fraction, % | 6.0% | 548/9190 |
| Blood(hematology, research) | Immature reticulocyte fraction, % | 97.7% | 8979/9190 |
| Blood(hematology, research) | Lymphocytes, % | 97.7% | 8977/9190 |
| Blood(hematology, research) | Macro red blood cell ratio, % | 97.7% | 8981/9190 |
| Blood(hematology, research) | Medicum flourescence ratio of reticulocytes | 97.7% | 8980/9190 |
| Blood(hematology, research) | Micro red blood cell ratio, % | 97.7% | 8981/9190 |
| Blood(hematology, research) | Monocytes, % | 97.7% | 8977/9190 |
| Blood(hematology, research) | Neutrophilocytes, % | 97.7% | 8975/9190 |
| Blood(hematology, research) | Nucleated red blood cell | 97.7% | 8982/9190 |
| Blood(hematology, research) | Nucleated red blood cell, % | 97.7% | 8982/9190 |
| Blood(hematology, research) | Red cell distribution width (standard deviation) | 97.7% | 8980/9190 |
| Blood(hematology, research) | Reticulocytes, % | 97.7% | 8977/9190 |
| Blood(hematology, research) | Reticulocyte hemoglobin equivalent | 97.7% | 8981/9190 |
| Blood(hematology, research) | Basophilocytes, % | 97.7% | 8980/9190 |
| Blood(hematology, research) | Delta hemoglobin equivalent of reticulocytes | 97.7% | 8981/9190 |
| Blood(hematology, research) | High flourescence ratio of reticulocytes | 97.7% | 8979/9190 |
| Blood(hematology, research) | Hyper-He%, RBC RET scattergram, forward, ratio | 97.7% | 8981/9190 |
| Blood(hematology, research) | Hypo-He%, RBC RET scattergram, forward, ratio | 97.7% | 8981/9190 |
| Blood(hematology, research) | Eosinophilocytes, % | 97.7% | 8978/9190 |
| Blood | Glomerular filtration; vol.rate(CKD-EPI) | 99.3% | 9124/9190 |
| Blood | Lactate (Flouride/oxa-tube) | 98.2% | 9029/9190 |
| Blood | 3-Hydroxybutyrate | 99.4% | 9136/9190 |
| Blood | Alanine transaminase | 99.4% | 9132/9190 |
| Blood | Albumin | 99.6% | 9155/9190 |
| Blood | Alkaline phosphatase | 99.4% | 9138/9190 |
| Blood | Amylase, pancreatic type | 99.4% | 9138/9190 |
| Blood | Antitrombin (enz.) | 99.3% | 9128/9190 |
| Blood | Alpha-1-Antitrypsin | 99.5% | 9142/9190 |
| Blood | Aspartate transaminase | 93.2% | 8569/9190 |
| Blood | Bilirubins | 99.6% | 9153/9190 |
| Blood | Calcium(II) | 99.6% | 9151/9190 |
| Blood | Chloride | 99.5% | 9146/9190 |
| Blood | Cholesterol+ester | 99.4% | 9133/9190 |
| Blood | Cholesterol+ester, in HDL | 99.4% | 9137/9190 |
| Blood | Cholesterol+ester, in LDL | 98.9% | 9087/9190 |
| Blood | Carbon dioxide, mmol/L | 99.4% | 9134/9190 |
| Blood | Coagulation, surface-induced, APTT | 99.0% | 9095/9190 |
| Blood | Coagulation, tissue factor-induced; INR | 99.3% | 9125/9190 |
| Blood | Cortisol | 99.4% | 9138/9190 |
| Blood | C-reactive protein [CRP] | 99.6% | 9155/9190 |
| Blood | Creatine kinase | 99.1% | 9110/9190 |
| Blood | Creatininium(enz) | 99.6% | 9154/9190 |
| Blood | Cystacin C | 99.5% | 9145/9190 |
| Blood | Ethanol | 99.2% | 9120/9190 |
| Blood | Ferritin | 99.1% | 9111/9190 |
| Blood | Fibrin D-Dimer | 99.3% | 9129/9190 |
| Blood | Fibrinogen | 99.2% | 9120/9190 |
| Blood | Folate | 97.1% | 8923/9190 |
| Blood | Glucose | 98.7% | 9069/9190 |
| Blood | Haemoglobin A1c | 99.1% | 9103/9190 |
| Blood | Lactate | 99.4% | 9135/9190 |
| Blood | Lactate dehydrogenase | 91.6% | 8422/9190 |
| Blood | Magnesium(II) | 99.5% | 9144/9190 |
| Blood | Myoglobin | 99.6% | 9149/9190 |
| Blood | Orosomucoid | 99.5% | 9141/9190 |
| Blood | Parathyrin hormone | 98.7% | 9071/9190 |
| Blood | Fructosamine | 98.3% | 9036/9190 |
| Blood | Gamma-Glutamyltransferase | 99.2% | 9121/9190 |
| Blood | Haptoglobin | 99.3% | 9122/9190 |
| Blood | Immunoglobulin A | 99.5% | 9140/9190 |
| Blood | Immunoglobulin G | 99.5% | 9140/9190 |
| Blood | Immunoglobulin M | 99.5% | 9140/9190 |
| Blood | Interleukin-6 | 99.0% | 9095/9190 |
| Blood | Iron(III) | 99.2% | 9120/9190 |
| Blood | Potassium ion | 99.3% | 9126/9190 |
| Blood | Phosphate(inorganic) | 99.4% | 9133/9190 |
| Blood | Protein | 99.4% | 9138/9190 |
| Blood | Sodium ion | 99.6% | 9154/9190 |
| Blood | Thyrotropin [TSH] | 99.6% | 9149/9190 |
| Blood | Thyroxine | 99.5% | 9148/9190 |
| Blood | Thyroxine binding globulin | 99.5% | 9146/9190 |
| Blood | Thyroxine (free) | 99.6% | 9154/9190 |
| Blood | Transferrin | 99.5% | 9144/9190 |
| Blood | Transferrinreceptor fragment | 99.5% | 9140/9190 |
| Blood | Triacylglycerol lipase | 99.5% | 9142/9190 |
| Blood | Triglyceride | 99.4% | 9132/9190 |
| Blood | Triiodothyronine | 99.5% | 9147/9190 |
| Blood | Triiodothyronine (free) | 99.6% | 9154/9190 |
| Blood | Troponine T | 99.1% | 9109/9190 |
| Blood | Urate | 99.5% | 9143/9190 |
| Blood | Urea | 95.3% | 8760/9190 |
| Blood | Cobalamin | 99.5% | 9148/9190 |
| Blood | Zinc | 98.6% | 9064/9190 |
| Blood(gas tension and acid/base) | O2 saturation, blood | 96.8% | 8895/9190 |
| Blood(gas tension and acid/base) | Base excess | 96.7% | 8886/9190 |
| Blood(gas tension and acid/base) | Haemoglobin(Fe) | 96.8% | 8895/9190 |
| Blood(gas tension and acid/base) | Hydrogen carbonate | 96.7% | 8886/9190 |
| Blood(gas tension and acid/base) | Calcium ion(free) | 96.9% | 8904/9190 |
| Blood(gas tension and acid/base) | Calcium ion(free) (pH=7.4) | 95.6% | 8786/9190 |
| Blood(gas tension and acid/base) | Carbon monoxide haemoglobin(Fe) | 96.8% | 8895/9190 |
| Blood(gas tension and acid/base) | Chloride | 99.5% | 9146/9190 |
| Blood(gas tension and acid/base) | Creatininium(enz) | 96.2% | 8841/9190 |
| Blood(gas tension and acid/base) | Glucose | 96.9% | 8905/9190 |
| Blood(gas tension and acid/base) | Lactate | 97.2% | 8934/9190 |
| Blood(gas tension and acid/base) | Methaemoglobin(Fe) | 96.8% | 8895/9190 |
| Blood(gas tension and acid/base) | Oxyhaemoglobin(Fe) | 96.8% | 8894/9190 |
| Blood(gas tension and acid/base) | Carbon dioxide, tension | 96.9% | 8909/9190 |
| Blood(gas tension and acid/base) | Hydrogen ion, pH | 96.9% | 8906/9190 |
| Blood(gas tension and acid/base) | Oxygen, tension | 96.9% | 8904/9190 |
| Blood(gas tension and acid/base) | Potassium ion | 96.9% | 8906/9190 |
| Blood(gas tension and acid/base) | Sodium ion | 96.9% | 8906/9190 |
| Blood(expensive) | Neutrophil gelatinase-associated lipocalin | 99.4% | 9134/9190 |
| Blood(expensive) | Proadrenomedullin (45-92) | 98.7% | 9073/9190 |
| Blood(expensive) | Pro-brain natriuretic peptide(1-76) | 99.5% | 9145/9190 |
| Blood(expensive) | Vasopressin-neurophysin 2-copeptin(126-164) | 98.8% | 9084/9190 |
| Blood(expensive) | Corticotropin | 98.2% | 9025/9190 |
| Blood(expensive) | Procalcitonin | 99.0% | 9096/9190 |
| Blood(expensive) | Urokinase plasminogen activator surface receptor(soluble) | 99.2% | 9112/9190 |
| Blood(hematology) | Basophilocytes | 99.4% | 9136/9190 |
| Blood(hematology) | Eosinophilocytes | 99.4% | 9136/9190 |
| Blood(hematology) | Ercs(B)-Haemoglobin (MCHC) | 99.3% | 9126/9190 |
| Blood(hematology) | Erythrocyte volumes; relative distribution width | 99.2% | 9119/9190 |
| Blood(hematology) | Erythrocytes; entitic vol (MCV) | 99.3% | 9125/9190 |
| Blood(hematology) | Erythrocytes | 99.5% | 9140/9190 |
| Blood(hematology) | Erythrocytes, vol.fr. | 99.3% | 9124/9190 |
| Blood(hematology) | Haemoglobin(Fe) | 99.4% | 9139/9190 |
| Blood(hematology) | Haemoglobin(Fe);B(Ercs) (MCH) | 99.4% | 9136/9190 |
| Blood(hematology) | Leukocytes | 99.5% | 9140/9190 |
| Blood(hematology) | Lymphocytes | 99.4% | 9137/9190 |
| Blood(hematology) | Metamyelo.+Myelo.+Promyelocytes | 97.7% | 8982/9190 |
| Blood(hematology) | Monocytes | 99.4% | 9136/9190 |
| Blood(hematology) | Neutrophilocytes | 99.4% | 9139/9190 |
| Blood(hematology) | Thrombocytes average volume | 98.3% | 9032/9190 |
| Blood(hematology) | Thrombocytes | 95.7% | 8797/9190 |
| Blood(hematology) | Platelets distribution width (PDW) | 98.3% | 9032/9190 |
| Blood(hematology) | Reticulocytes | 99.5% | 9140/9190 |
| Diagnosis | Charlson index 5 years, 1987 version | 100.0% | 9190/9190 |
| Diagnosis | Charlson index 5 years, 2011 version | 100.0% | 9190/9190 |
| Diagnosis | # days ago diagnosis: Chronic pulmonary disease was last seen within 5 years | 17.2% | 1585/9190 |
| Diagnosis | # days ago diagnosis: Intoxication was last seen within 5 years | 4.2% | 382/9190 |
| Diagnosis | # days ago diagnosis: Gastroenterology was last seen within 5 years | 14.0% | 1286/9190 |
| Diagnosis | # days ago diagnosis: Chest pain was last seen within 5 years | 9.6% | 882/9190 |
| Diagnosis | # days ago diagnosis: Kidney insufficiency was last seen within 5 years | 6.8% | 623/9190 |
| Diagnosis | # days ago diagnosis: Syncope and malaise was last seen within 5 years | 13.6% | 1254/9190 |
| Diagnosis | # days ago diagnosis: COVID19 was last seen within 5 years | 1.1% | 102/9190 |
| Diagnosis | # days ago diagnosis: Other infection was last seen within 5 years | 6.0% | 554/9190 |
| Diagnosis | # days ago diagnosis: Acute alcohol consumption was last seen within 5 years | 4.2% | 387/9190 |
| Diagnosis | # days ago diagnosis: Chronic alcohol consumption was last seen within 5 years | 7.8% | 715/9190 |
| Diagnosis | # days ago diagnosis: Electrolyte-problems was last seen within 5 years | 11.3% | 1042/9190 |
| Diagnosis | # days ago diagnosis: Urinary tract infection (UTI) was last seen within 5 years | 13.6% | 1250/9190 |
| Diagnosis | # days ago diagnosis: Pneumonia was last seen within 5 years | 19.3% | 1775/9190 |
| Diagnosis | # days ago diagnosis: Erysipelas was last seen within 5 years | 3.2% | 292/9190 |
| Diagnosis | # days ago diagnosis: Sepsis was last seen within 5 years | 5.6% | 512/9190 |
| Diagnosis | Number of previous group 1 diagnosis observed in last 5 years | 17.2% | 1585/9190 |
| Diagnosis | Number of previous group 10 diagnosis observed in last 5 years | 4.2% | 382/9190 |
| Diagnosis | Number of previous group 12 diagnosis observed in last 5 years | 14.0% | 1286/9190 |
| Diagnosis | Number of previous group 13 diagnosis observed in last 5 years | 9.6% | 882/9190 |
| Diagnosis | Number of previous group 14 diagnosis observed in last 5 years | 6.8% | 623/9190 |
| Diagnosis | Number of previous group 15 diagnosis observed in last 5 years | 13.6% | 1254/9190 |
| Diagnosis | Number of previous group 16 diagnosis observed in last 5 years | 1.1% | 102/9190 |
| Diagnosis | Number of previous group 17 diagnosis observed in last 5 years | 6.0% | 554/9190 |
| Diagnosis | Number of previous group 2 diagnosis observed in last 5 years | 4.2% | 387/9190 |
| Diagnosis | Number of previous group 3 diagnosis observed in last 5 years | 7.8% | 715/9190 |
| Diagnosis | Number of previous group 4 diagnosis observed in last 5 years | 11.3% | 1042/9190 |
| Diagnosis | Number of previous group 5 diagnosis observed in last 5 years | 13.6% | 1250/9190 |
| Diagnosis | Number of previous group 6 diagnosis observed in last 5 years | 19.3% | 1775/9190 |
| Diagnosis | Number of previous group 7 diagnosis observed in last 5 years | 3.2% | 292/9190 |
| Diagnosis | Number of previous group 8 diagnosis observed in last 5 years | 5.6% | 512/9190 |
| ECG | P-displacement | 85.0% | 7807/9190 |
| ECG | PR interval (ms) | 84.7% | 7781/9190 |
| ECG | P-start | 85.0% | 7807/9190 |
| ECG | QRS-forskydning | 97.1% | 8926/9190 |
| ECG | QRS-varighed (ms) | 97.1% | 8926/9190 |
| ECG | Q-start | 97.1% | 8926/9190 |
| ECG | Q-T interval (ms) | 97.1% | 8926/9190 |
| ECG | QTc (Bazett) | 97.1% | 8926/9190 |
| ECG | QTc Framingham | 97.1% | 8926/9190 |
| ECG | QTc Fridericia | 97.1% | 8926/9190 |
| ECG | R-akse | 97.1% | 8926/9190 |
| ECG | AC interference | 97.1% | 8926/9190 |
| ECG | ECG recognition | 97.1% | 8926/9190 |
| ECG | Atrial heart rate | 97.1% | 8926/9190 |
| ECG | Baseline change | 97.1% | 8926/9190 |
| ECG | Lead error | 97.1% | 8926/9190 |
| ECG | Lead quality | 97.1% | 8926/9190 |
| ECG | Lead saturation | 97.1% | 8926/9190 |
| ECG | Electrode noise | 97.1% | 8926/9190 |
| ECG | Muscle artifact | 97.1% | 8926/9190 |
| ECG | Number of QRS complexes | 97.1% | 8926/9190 |
| ECG | P-axis | 83.6% | 7682/9190 |
| ECG | T-axis | 97.1% | 8926/9190 |
| ECG | T-displacement | 97.1% | 8926/9190 |
| ECG | T-start | 97.1% | 8926/9190 |
| ECG | ventricular heart rate | 97.1% | 8926/9190 |
| Nurse(physical measures) | Calculated BMI | 59.3% | 5450/9190 |
| Nurse(physical measures) | Diastolic blood pressure | 99.1% | 9111/9190 |
| Nurse(physical measures) | Height | 67.1% | 6165/9190 |
| Nurse(physical measures) | Pulse rate | 99.2% | 9114/9190 |
| Nurse(physical measures) | Respiratory rate | 99.1% | 9108/9190 |
| Nurse(physical measures) | Oxygen saturation, monitored | 99.2% | 9119/9190 |
| Nurse(physical measures) | systolic blood pressure | 99.2% | 9113/9190 |
| Nurse(physical measures) | Temperature 0C | 96.8% | 8892/9190 |
| Nurse(physical measures) | Weight | 66.7% | 6126/9190 |
| Nurse(patient scores) | Critical score (Aggregate weighted scoring system: TOKS) | 79.2% | 7275/9190 |
| Nurse(patient scores) | Glasgow Coma scale | 98.0% | 9006/9190 |
| Nurse(patient scores) | Level of consciousness | 97.7% | 8977/9190 |
| Nurse(patient scores) | Triage color | 95.0% | 8731/9190 |
| Urine(dipsticks) | U-pH | 55.2% | 5073/9190 |
| Urine(dipsticks) | U-blood | 40.6% | 3732/9190 |
| Urine(dipsticks) | U-Glucose | 55.2% | 5072/9190 |
| Urine(dipsticks) | U-ketone | 55.3% | 5082/9190 |
| Urine(dipsticks) | U-leukocytes | 55.3% | 5079/9190 |
| Urine(dipsticks) | U-nitrite | 55.3% | 5085/9190 |
| Urine(dipsticks) | U-Protein | 54.2% | 4983/9190 |
| Person | Age | 100.0% | 9190/9190 |
| Person | Gender | 100.0% | 9190/9190 |
| PrevAdm | #days since last admittance | 69.8% | 6414/9190 |
| Urine(biochem+Flow) | Albumin, urine | 39.1% | 3593/9190 |
| Urine(biochem+Flow) | Bacteria, urine | 37.9% | 3481/9190 |
| Urine(biochem+Flow) | Creatininium, urine | 96.2% | 8841/9190 |
| Urine(biochem+Flow) | Erythrocytes, urine | 37.3% | 3428/9190 |
| Urine(Flow, research) | SF-forward scatter intensity | 8.8% | 809/9190 |
| Urine(Flow, research) | SF-forward scatter light width | 8.8% | 809/9190 |
| Urine(Flow, research) | Large red blood cells | 37.3% | 3424/9190 |
| Urine(Flow, research) | Non-lysed red blood cells | 0.0% | 0/9190 |
| Urine(Flow, research) | Lysed red blood cells | 37.3% | 3425/9190 |
| Urine(Flow, research) | White blood cells | 37.3% | 3425/9190 |
| Urine(Flow, research) | White blood cell clumps | 37.3% | 3425/9190 |
| Urine(Flow, research) | Epithelial cells | 37.3% | 3425/9190 |
| Urine(Flow, research) | Squamous epithelial cells | 37.3% | 3425/9190 |
| Urine(Flow, research) | Non-squamous epithelial cells | 37.3% | 3425/9190 |
| Urine(Flow, research) | Transitional epithelial cells | 37.3% | 3425/9190 |
| Urine(Flow, research) | Renal tubular epithelial cells | 37.3% | 3425/9190 |
| Urine(Flow, research) | Atypical cells | 37.3% | 3425/9190 |
| Urine(Flow, research) | Casts | 37.3% | 3425/9190 |
| Urine(Flow, research) | Hyaline casts | 37.3% | 3425/9190 |
| Urine(Flow, research) | Non-hyaline casts | 37.3% | 3425/9190 |
| Urine(Flow, research) | Crystals | 37.3% | 3425/9190 |
| Urine(Flow, research) | Yeast-like cells | 37.3% | 3425/9190 |
| Urine(Flow, research) | Spermatozoa | 37.3% | 3425/9190 |
| Urine(Flow, research) | Mucus | 37.3% | 3425/9190 |
| Urine(Flow, research) | Conductivity | 37.3% | 3425/9190 |
| Urine(Flow, research) | Osmolality | 37.3% | 3425/9190 |
| Urine(Flow, research) | TC-SF | 37.2% | 3421/9190 |
| Urine(Flow, research) | TC-CW | 37.2% | 3421/9190 |
| Urine(Flow, research) | TC-CB | 37.3% | 3425/9190 |
| Urine(Flow, research) | Others-SF | 37.2% | 3420/9190 |
| Urine(Flow, research) | Others-CW | 37.3% | 3425/9190 |
| Urine(Flow, research) | Debris | 37.3% | 3425/9190 |
| Urine(Flow, correlated) | Small red blood cells | 37.3% | 3425/9190 |
| Blood(hematology, correlated) | Platelet large cell ratio | 96.5% | 8864/9190 |
| Blood(hematology, correlated) | Plateletcrit, % | 96.5% | 8865/9190 |
| Blood(hematology, correlated) | Platelets-impedance | 97.6% | 8974/9190 |
| Blood(hematology, correlated) | Total nucleated cell absolute count | 97.7% | 8982/9190 |
| Blood(hematology, correlated) | White blood cells absolute count-WNR | 97.7% | 8982/9190 |
| Blood(hematology, correlated) | Total nucleated cell absolute count–WNR | 97.7% | 8982/9190 |
| Blood(hematology, correlated) | Basophil absolute count – WNR channel | 97.7% | 8982/9190 |
| Blood(hematology, correlated) | White blood cell absolute count-WDF | 97.7% | 8981/9190 |
| Blood(hematology, correlated) | Total nucleated cell absolute count-WDF | 97.7% | 8981/9190 |
| Blood(hematology, correlated) | Lymphocyte absolute count | 97.7% | 8981/9190 |
| Blood(hematology, correlated) | Red blood cells-RET | 97.7% | 8981/9190 |
| Blood(hematology, correlated) | Platelets-optical | 97.7% | 8980/9190 |
| Blood(hematology, correlated) | Red blood cells-hemoglobin equivalent | 97.7% | 8980/9190 |
| Blood(hematology, correlated) | Mature red blood cells-RET | 97.7% | 8980/9190 |
| Blood(hematology, correlated) | Reticulocyte produkction index | 97.7% | 8978/9190 |
| Blood(hematology, correlated) | Total nucleated cell absolute count-RET | 97.7% | 8981/9190 |
| Blood(hematology, correlated) | Platelets-flourescence-F1 | 6.0% | 548/9190 |
| Blood(hematology, correlated) | Hemoglobin-RET | 97.7% | 8981/9190 |
| Blood(hematology, correlated) | Platelets-flourescence-F2 | 6.0% | 548/9190 |
| Blood(hematology, correlated) | Mean cell haemoglobin concentration-RET | 97.7% | 8980/9190 |
| Blood(gas tension and acid/base, research) | Total CO2 | 96.0% | 8822/9190 |
| Blood(gas tension and acid/base, research) | Anion gap (K+) | 96.1% | 8834/9190 |
| Blood(gas tension and acid/base, research) | p50(actual) | 96.0% | 8818/9190 |
| Blood(gas tension and acid/base, research) | Anion gap | 96.1% | 8834/9190 |
| Blood(gas tension and acid/base, research) | p50(standard) | 96.0% | 8818/9190 |

Supplemental table S2

This table indicates that the patient is included in the noted diagnosis group if the ICD-10 code was present unless there was also used an ICD-10 code listed as diagnosis group discard.

| ICD-10 code | Description | Diagnosis group | Diagnosis group Discard |
| --- | --- | --- | --- |
| J44.0 | Chronic obstructive pulmonary disease with acute lower respiratory infection | Chronic pulmonary disease |  |
| J44.1 | Chronic obstructive pulmonary disease with acute exacerbation, unspecified | Chronic pulmonary disease |  |
| J44.8 | Other specified chronic obstructive pulmonary disease | Chronic pulmonary disease |  |
| J44.9 | Chronic obstructive pulmonary disease, unspecified | Chronic pulmonary disease |  |
| J45 | Asthma | Chronic pulmonary disease |  |
| J45.0 | Predominantly allergic asthma | Chronic pulmonary disease |  |
| J45.1 | Nonallergic asthma | Chronic pulmonary disease |  |
| J45.8 | Mixed asthma | Chronic pulmonary disease |  |
| J45.9 | Asthma, unspecified | Chronic pulmonary disease |  |
| J46 | Status asthmaticus | Chronic pulmonary disease |  |
| C34.9 | Malignant neoplasm of unspecified part of bronchus or lung |  | Chronic pulmonary disease |
| J90.9 | Pleural effusion, not elsewhere classified |  | Chronic pulmonary disease |
| J96.0 | Acute respiratory failure |  | Chronic pulmonary disease |
| J96.1 | Chronic respiratory failure |  | Chronic pulmonary disease |
| J96.9 | Respiratory failure, unspecified |  | Chronic pulmonary disease |
| R05.9 | Cough, unspecified |  | Chronic pulmonary disease |
| R06.0 | Dyspnoea |  | Chronic pulmonary disease |
| R06.4 | Hyperventilation |  | Chronic pulmonary disease |
| F10.0 | Acute intoxication, ethanol | Acute alcohol consumption |  |
| T51.0 | Toxic effect of ethanol | Acute alcohol consumption |  |
| T51.9 | Toxic effect of alcohol, unspecified | Acute alcohol consumption |  |
| F10.1 | Harmful use, ethanol | Chronic alcohol consumption |  |
| F10.2 | Dependence syndrome, ethanol | Chronic alcohol consumption |  |
| F10.3 | Withdrawal state, ethanol | Chronic alcohol consumption |  |
| F10.4 | Withdrawal state with delirium, ethanol | Chronic alcohol consumption |  |
| K70.0 | Alcoholic fatty liver | Chronic alcohol consumption |  |
| K70.1 | Alcoholic hepatitis | Chronic alcohol consumption |  |
| K70.2 | Alcoholic fibrosis and sclerosis of liver | Chronic alcohol consumption |  |
| K70.3 | Alcoholic cirrhosis of liver | Chronic alcohol consumption |  |
| K70.4 | Alcoholic hepatic failure | Chronic alcohol consumption |  |
| K70.9 | Alcoholic liver disease, unspecified | Chronic alcohol consumption |  |
| K86.0 | Alcohol-induced chronic pancreatitis | Chronic alcohol consumption |  |
| R18 | Ascites | Chronic alcohol consumption |  |
| N10 | Acute tubulo-interstitial nephritis | UTI (Urinary tract infection) |  |
| N11.8 | Other chronic tubulo-interstitial nephritis | UTI (Urinary tract infection) |  |
| N12.9 | Tubulo-interstitial nephritis, not specified as acute or chronic | UTI (Urinary tract infection) |  |
| N30 | Cystitis | UTI (Urinary tract infection) |  |
| N30.0 | Acute cystitis | UTI (Urinary tract infection) |  |
| N30.8 | Other cystitis | UTI (Urinary tract infection) |  |
| N30.9 | Cystitis, unspecified | UTI (Urinary tract infection) |  |
| N39.0 | Urinary tract infection, site not specified | UTI (Urinary tract infection) |  |
| A31.0A | Pulmonary mycobacterial infection | Pneumonia |  |
| A48.1 | Legionnaires disease | Pneumonia |  |
| J13 | Pneumonia due to Streptococcus pneumoniae | Pneumonia |  |
| J14 | Pneumonia due to Haemophilus influenzae | Pneumonia |  |
| J15 | Bacterial pneumonia, not elsewhere classified | Pneumonia |  |
| J15.0 | Pneumonia due to Klebsiella pneumoniae | Pneumonia |  |
| J15.1 | Pneumonia due to Pseudomonas | Pneumonia |  |
| J15.3 | Pneumonia due to streptococcus, group B | Pneumonia |  |
| J15.7 | Pneumonia due to Mycoplasma pneumoniae | Pneumonia |  |
| J15.8 | Other bacterial pneumonia | Pneumonia |  |
| J15.9 | Bacterial pneumonia, unspecified | Pneumonia |  |
| J18 | Pneumonia, organism unspecified | Pneumonia |  |
| J18.1 | Lobar pneumonia, unspecified | Pneumonia |  |
| J18.9 | Pneumonia, unspecified | Pneumonia |  |
| J86.9 | Pyothorax without fistula | Pneumonia |  |
| A46 | Erysipelas | Erysipelas |  |
| A32.7 | Listerial sepsis | Sepsis |  |
| A40.2 | Sepsis due to streptococcus, group D and enterococcus | Sepsis |  |
| A40.3 | Sepsis due to Streptococcus pneumoniae | Sepsis |  |
| A40.8 | Other streptococcal sepsis | Sepsis |  |
| A40.9 | Streptococcal sepsis, unspecified | Sepsis |  |
| A41.0 | Sepsis due to Staphylococcus aureus | Sepsis |  |
| A41.2 | Sepsis due to unspecified staphylococcus | Sepsis |  |
| A41.5 | Sepsis due to other Gram-negative organisms | Sepsis |  |
| A41.8 | Other specified sepsis | Sepsis |  |
| A41.9 | Sepsis, unspecified | Sepsis |  |
| A49.9A | Bacterial infection, unspecified | Sepsis |  |
| R57.2 | Septic shock | Sepsis |  |
| F14.0 | Mental and behavioural disorders due to use of cocaine | Intoxication |  |
| F15.0 | Mental and behavioural disorders due to use of other stimulants, including caffeine | Intoxication |  |
| F15.50 | Mental and behavioural disorders due to use of other stimulants, including caffeine, psychotic disorder | Intoxication |  |
| F16.0 | Mental and behavioural disorders due to use of hallucinogens | Intoxication |  |
| F19.00 | Mental and behavioural disorders due to multiple drug use and use of other psychoactive substances | Intoxication |  |
| T39.0 | Salicylates poisioning | Intoxication |  |
| T39.8A | Acetaminophen poisioning | Intoxication |  |
| T39.9 | Nonopioid analgesic, antipyretic and antirheumatic, unspecified | Intoxication |  |
| T40.0 | Opium poisioning | Intoxication |  |
| T40.1 | Heroin poisioning | Intoxication |  |
| T40.2 | Other opioid poisioning | Intoxication |  |
| T40.2B | Morphine poisioning | Intoxication |  |
| T40.3 | Methadone poisioning | Intoxication |  |
| T40.4 | Other synthetic narcotics poisioning | Intoxication |  |
| T40.5 | Cocaine poisioning | Intoxication |  |
| T40.6 | Other and unspecified narcotics poisioning | Intoxication |  |
| T40.7 | Cannabis (derivatives) poisioning | Intoxication |  |
| T40.9 | Other and unspecified psychodysleptics [hallucinogens] poisioning | Intoxication |  |
| T42.4 | Benzodiazepines poisioning | Intoxication |  |
| T42.6 | Other antiepileptic and sedative-hypnotic drugs poisioning | Intoxication |  |
| T42.7 | Antiepileptic and sedative-hypnotic drugs, unspecified poisioning | Intoxication |  |
| T43.2 | Other and unspecified antidepressants poisioning | Intoxication |  |
| T43.5 | Other and unspecified antipsychotics and neuroleptics poisioning | Intoxication |  |
| T43.6 | Psychostimulants with abuse potential poisioning | Intoxication |  |
| T44.0 | Poisoning by drugs primarily affecting the autonomic nervous system, Anticholinesterase agents | Intoxication |  |
| T44.9 | Poisoning by drugs primarily affecting the autonomic nervous system, other and unspecified drugs primarily affecting the autonomic nervous system | Intoxication |  |
| T45.0A | Poisoning by primarily systemic and haematological agents, not elsewhere classified, Antiallergic and antiemetic drugs | Intoxication |  |
| T45.9A | Poisoning by primarily systemic and haematological agents, not elsewhere classified, Primarily systemic and haematological agent, unspecified | Intoxication |  |
| T46.0 | Poisoning by agents primarily affecting the cardiovascular system, Cardiac-stimulant glycosides and drugs of similar action | Intoxication |  |
| T49.9 | Poisoning by topical agents primarily affecting skin and mucous membrane and by ophthalmological, otorhinolaryngological and dental drugs, Topical agent, unspecified | Intoxication |  |
| T50.0 | Poisoning by diuretics and other and unspecified drugs, medicaments and biological substances, Mineralocorticoids and their antagonists | Intoxication |  |
| T50.9 | Poisoning by diuretics and other and unspecified drugs, medicaments and biological substances, Other and unspecified drugs, medicaments and biological substances | Intoxication |  |
| T51.1 | Toxic effect of alcohol, methanol | Intoxication |  |
| T59.9 | Toxic effect of other gases, fumes and vapours, Gases, fumes and vapours, unspecified | Intoxication |  |
| T62.0 | Toxic effect of other noxious substances eaten as food, Ingested mushrooms | Intoxication |  |
| X67.10 | Intentional self-poisoning by and exposure to carbon monoxide from utility gas | Intoxication |  |
| A02.9 | Salmonella infection, unspecified | Gastroenterology |  |
| A04.5 | Other bacterial intestinal infections, Campylobacter enteritis | Gastroenterology |  |
| A04.7 | Other bacterial intestinal infections, Enterocolitis due to Clostridium difficile | Gastroenterology |  |
| A07.1 | Other protozoal intestinal diseases, Giardiasis | Gastroenterology |  |
| A08.0 | Viral and other specified intestinal infections, Rotaviral enteritis | Gastroenterology |  |
| A08.1 | Viral and other specified intestinal infections, Acute gastroenteropathy due to Norovirus | Gastroenterology |  |
| A09.0 | Other gastroenteritis and colitis of infectious and unspecified origin, Other and unspecified gastroenteritis and colitis of infectious origin | Gastroenterology |  |
| A09.9 | Other gastroenteritis and colitis of infectious and unspecified origin, Gastroenteritis and colitis of unspecified origin | Gastroenterology |  |
| K52.9B | Noninfective gastroenteritis and colitis, unspecified | Gastroenterology |  |
| K56.6C | Other and unspecified intestinal obstruction | Gastroenterology |  |
| K56.7 | Ileus, unspecified | Gastroenterology |  |
| K59.0 | Constipation | Gastroenterology |  |
| K80.0 | Calculus of gallbladder with acute cholecystitis | Gastroenterology |  |
| K80.1 | Calculus of gallbladder with other cholecystitis | Gastroenterology |  |
| K80.3 | Calculus of bile duct with cholangitis | Gastroenterology |  |
| K80.4 | Calculus of bile duct with cholecystitis | Gastroenterology |  |
| K81.0 | Acute cholecystitis | Gastroenterology |  |
| K81.9 | Cholecystitis, unspecified | Gastroenterology |  |
| K83.0 | Cholangitis | Gastroenterology |  |
| R11 | Nausea and vomiting | Gastroenterology |  |
| N17.9 | Acute renal failure, unspecified | Kidney insufficiency |  |
| N18.3 | Chronic kidney disease, stage 3 | Kidney insufficiency |  |
| N18.4 | Chronic kidney disease, stage 4 | Kidney insufficiency |  |
| N18.5 | Chronic kidney disease, stage 5 | Kidney insufficiency |  |
| N18.9 | Chronic kidney disease, unspecified | Kidney insufficiency |  |
| N19.9 | Unspecified kidney failure | Kidney insufficiency |  |
| N17.9 | Acute renal failure, unspecified |  |  |
| R42 | Dizziness and giddiness | Syncope and malaise |  |
| R53 | Malaise and fatigue | Syncope and malaise |  |
| R55 | Syncope and collapse | Syncope and malaise |  |
| R29.6 | Tendency to fall, not elsewhere classified |  | Syncope and malaise |
| R41.0 | Disorientation, unspecified |  | Syncope and malaise |
| B34.2 | Coronavirus infection, unspecified site | COVID19 |  |
| B97.2 | Coronavirus as the cause of diseases classified to other chapters | COVID19 |  |
| A49.0 | Staphylococcal infection, unspecified site | Other infection |  |
| A49.1 | Streptococcal and enterococcal infection, unspecified site | Other infection |  |
| A49.2 | Haemophilus influenzae infection, unspecified site | Other infection |  |
| A49.8 | Other bacterial infections of unspecified site | Other infection |  |
| A49.9 | Bacterial infection, unspecified | Other infection |  |
| A54.0 | Gonococcal infection of lower genitourinary tract without periurethral or accessory gland abscess | Other infection |  |
| A56.0 | Chlamydial infection of lower genitourinary tract | Other infection |  |
| B95.4 | Other streptococcus as the cause of diseases classified to other chapters | Other infection |  |
| B95.6 | Staphylococcus aureus as the cause of diseases classified to other chapters | Other infection |  |
| B96.1 | Klebsiella pneumoniae [K. pneumoniae] as the cause of diseases classified to other chapters | Other infection |  |
| B96.2 | Escherichia coli [E. coli] as the cause of diseases classified to other chapters | Other infection |  |
| B96.3 | Haemophilus influenzae [H. influenzae] as the cause of diseases classified to other chapters | Other infection |  |
| B96.5 | Pseudomonas (aeruginosa) as the cause of diseases classified to other chapters | Other infection |  |
| B98.0 | Helicobacter pylori [H.pylori] as the cause of diseases classified to other chapters | Other infection |  |
| G00.1 | Pneumococcal meningitis | Other infection |  |
| G00.9 | Bacterial meningitis, unspecified | Other infection |  |
| H60.0 | Abscess of external ear | Other infection |  |
| H60.9 | Otitis externa, unspecified | Other infection |  |
| H66.0 | Acute suppurative otitis media | Other infection |  |
| H66.1 | Chronic tubotympanic suppurative otitis media | Other infection |  |
| H66.3 | Other chronic suppurative otitis media | Other infection |  |
| I33.0 | Acute and subacute infective endocarditis | Other infection |  |
| J01.9 | Acute sinusitis, unspecified | Other infection |  |
| J03.0 | Streptococcal tonsillitis | Other infection |  |
| R07 | Pain in throat and chest | Chest pain |  |
| R07.3 | Other chest pain | Chest pain |  |
| R07.4 | Chest pain, unspecified | Chest pain |  |

Supplemental table S3
Decided limits for reporting panic values.

| **Analysis** | **Unit** | **Lower limit (number of alarm results)** | **Upper limit (number of alarm results)** |
| --- | --- | --- | --- |
|  |  |  |  |
|  |  |  |  |
| Antithrombin (enz.) | 10^3^ IU/L | 0.5 (21) |  |
| Alpha-1-Antitrypsin | g/L | 0.3 (3) |  |
| Calcium(II) | mmol/L | 0.8 (0) | 1.5 (0) |
| Carbon monoxide haemoglobin(Fe) | Fraction |  | 0.2 (2) |
| Ethanol | mmol/L |  | 83 (40) |
| Fibrin D-Dimer | mg/L FEU |  | 5 (445) |
| Fibrinogen (coag.) | µmol/L | 3 (2) |  |
| Haptoglobin | g/L | 0.2 (83) |  |
| Hydrogen carbonate | mmol/L | 10 (23) |  |
| Immunoglobulin A | g/L | 0.3 (37) | 7 (83) |
| Immunoglobulin G | g/L | 3 (9) | 25 (24) |
| Immunoglobulin M | g/L | 0.2 (94) | 5 (36) |
| Chloride | mmol/L | 80 (26) | 115 (10) |
| Coagulation, tissue factor-induced | seconds |  | 100 (0) |
| Cholesterol+ester, in HDL | mmol/L |  | 10 (5) |
| Cortisol | nmol/L | 50 (55) |  |
| Creatine kinase | U/L |  | 1000 (134) |
| Magnesium(II) | mmol/L | 0.3 (4) | 1.5 (6) |
| Methaemoglobin(Fe) | Fraction |  | 0.15 (0) |
| Thyrotropin [TSH] | 10-^3^IU/L | 0.05 (64) | 10 (48) |
| Thyroxine | pmol/L |  | 35 (11) |
| Triglyceride | mmol/L |  | 12 (8) |
| Triiodothyronine (free) | pmol/L | 1.5 (28) | 12 (6) |
| Troponine T | ng/L |  | 70 (532) |
| Cobalamin | pmol/L | 74 (2) |  |
| Zinc | µmol/l | 3 (33) |  |

Supplemental table S4

Standard list of biomarkers required at admission in the medical emergency department.

| Analysis name |
| --- |
| Alanine transaminase |
| Albumin |
| Alkaline phosphatase |
| Amylase, pancreatic type |
| Basophilocytes |
| Bilirubins |
| Calcium(II) |
| Coagulation, tissue factor-induced; INR |
| C-reactive protein |
| Creatininium(enz) |
| Eosinophilocytes |
| Glucose |
| Haemoglobin A1c |
| Haemoglobin(Fe) |
| Lactate |
| Lactate dehydrogenase |
| Leukocytes |
| Lymphocytes |
| Monocytes |
| Neutrophilocytes |
| Potassium ion |
| Sodium ion |
| Thrombocytes |
| Urea |
| ECG |
